# Osteoclasts control endochondral ossification via regulating acetyl-CoA availability

**DOI:** 10.1038/s41413-024-00360-6

**Published:** 2024-08-28

**Authors:** Daizhao Deng, Xianming Liu, Wenlan Huang, Sirui Yuan, Genming Liu, Shanshan Ai, Yijie Fu, Haokun Xu, Xinyi Zhang, Shihai Li, Song Xu, Xiaochun Bai, Yue Zhang

**Affiliations:** 1 Department of Cell Biology, School of Basic Medical Science, Southern Medical University, Guangzhou, 510515 Guangdong China; 2 Department of Physiology, School of Basic Medical Science, Southern Medical University, Guangzhou, 510515 Guangdong China; 3 Department of Orthopaedics, Nanfang Hospital, Southern Medical University, Guangzhou, 510515 Guangdong China

**Keywords:** Energy metabolism, Bone

## Abstract

Osteoclast is critical in skeletal development and fracture healing, yet the impact and underlying mechanisms of their metabolic state on these processes remain unclear. Here, by using osteoclast-specific small GTPase Rheb1-knockout mice, we reveal that mitochondrial respiration, rather than glycolysis, is essential for cathepsin K (CTSK) production in osteoclasts and is regulated by Rheb1 in a mechanistic target of rapamycin complex 1 (mTORC1)-independent manner. Mechanistically, we find that Rheb1 coordinates with mitochondrial acetyl-CoA generation to fuel CTSK, and acetyl-CoA availability in osteoclasts is the central to elevating CTSK. Importantly, our findings demonstrate that the regulation of CTSK by acetyl-CoA availability is critical and may confer a risk for abnormal endochondral ossification, which may be the main cause of poor fracture healing on alcohol consumption, targeting Rheb1 could successfully against the process. These findings uncover a pivotal role of mitochondria in osteoclasts and provide a potent therapeutic opportunity in bone disorders.

## Introduction

Osteoclasts are specialized cells that play a crucial role in shaping bone morphology and regulating bone quality in vertebrate skeletal system. To maintain bone matrix homeostasis during skeletal development and remodeling, osteoclasts must efficiently meet their energetic demands for bone resorption within their relatively short lifespan. To dissolve both the inorganic and organic components of the bone matrix, osteoclasts must adhere tightly to the bone surface. They form a ruffled border to facilitate the delivery of collagenolytic enzymes, gelatinase, and protons, enabling the degradation and resorption of the bone matrix.^1,2^ Different resorbed substrates necessitate specific osteoclast behaviors for their degradation.^3^ Thus, selecting appropriate metabolic pathways to fine-tune osteoclast state transitions is crucial for regulating resorption processes.

Glycolysis and mitochondrial energetics are the two primary metabolic pathways that convert nutrients into ATP, fueling fundamental biological processes.^4–7^ Osteoclasts process abundant mitochondria with high energy-generating capacity.^8^ Since their identification, few studies have addressed the physiological metabolic requirements of osteoclasts during bone resorption. Metabolic reprogramming in osteoclasts suggests that both glycolysis and mitochondrial respiration are essential for their function and activity, with a greater reliance on glycolysis.^9–12^ However, the specific metabolic requirements of bone-attached osteoclasts and the reliance of mature osteoclasts on these pathways to maintain function remain unclear.

The replacement of cartilage and remodeling of bone matrix during growth or repair require precise orchestration of osteoclast-derived enzymes.^13–16^ Osteoclast deficiency or malfunction can impair endochondral ossification, leading to skeletal deformities, short stature,^17–19^ and disrupted fracture repair.^20,21^ Notably, studies indicate that levels of cathepsin K (CTSK)-generated collagen fragments, C-terminal telopeptide of type I collagen (CTX-I),^22^ can be affected by food intake.^23–25^ Additionally, abnormal skeletal growth^26,27^ and bone remodeling^28–31^ have been linked to alcohol consumption, indicating a potential influence on osteoclast activity. These observations suggest that varying nutritional and metabolic environments may significantly affect osteoclast resorption behaviors. Therefore, a detailed understanding of metabolic regulation in resorbing osteoclasts is crucial.

Rheb1, a small GTPase ubiquitously expressed in mammalian cells,^32^ is an essential activator of the nutrient sensor mechanistic target of rapamycin complex 1 (mTORC1)^33,34^ and a regulator of cellular metabolism, participating in both glycolysis and oxidative phosphorylation (OxPhos).^35,36^ Using a mouse line with osteoclast-specific Rheb1 knockout (KO), we found that mitochondrial respiration in multinucleated osteoclasts, regulated by Rheb1, is essential for maintaining CTSK abundance. Rheb1 loss in osteoclasts attenuated mitochondrial respiration, diminished acetyl-CoA levels, reduced CTSK, and impaired collagen degradation. These effects led to restricted postnatal growth and defective bone fracture repair in mice. We demonstrated that mitochondrial respiration, rather than glycolysis, is crucial for procathepsin K production in osteoclasts, particularly for their behavior on collagen. Our ex vivo and in vivo evidence indicate that acetyl-CoA availability is crucial for fine-tuning osteoclast CTSK levels, with mitochondrial acetyl-CoA generation playing a central role in this regulation. Rheb1 regulates mitochondrial respiration, enabling osteoclasts to produce CTSK necessary for effective endochondral ossification. This understanding may provide opportunities to counteract osteoclast resorption function in diseases by targeting their metabolic pathways.

## Results

### Rheb1 deletion impairs osteoclast function, resulting in growth restriction and delayed bone fracture healing

**Fig. 1:**
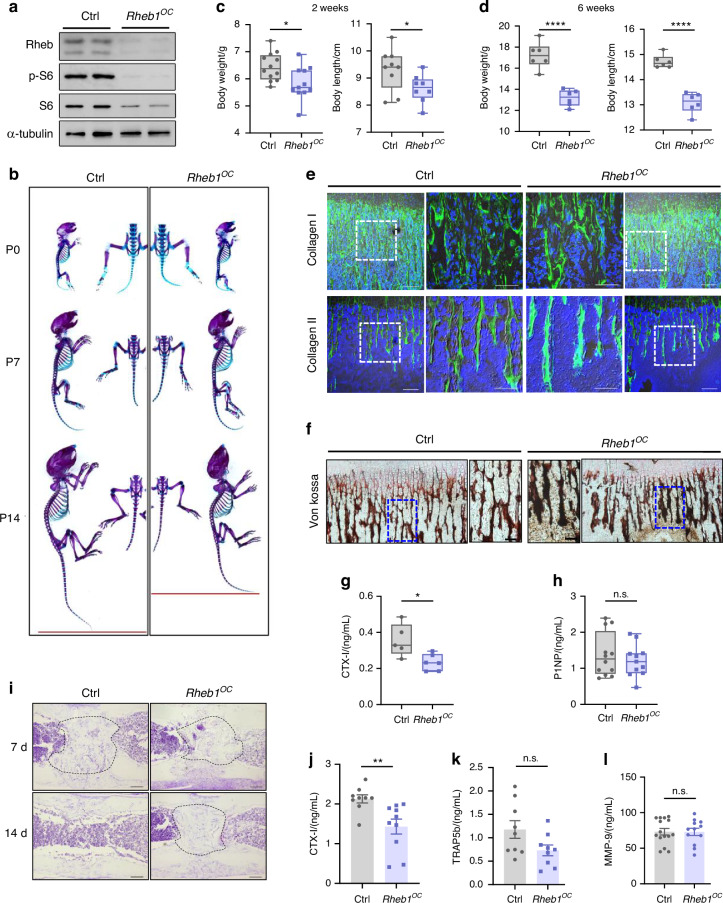
Rheb1 deletion impairs osteoclast function, resulting in growth restriction and delayed bone fracture healing. **a** Immunoblots of the Rheb1 level and mTORC1 activity (S6 and phosphorylated S6) in osteoclasts induced from *Rheb1*^*OC*^ or control mice BMDMs, with ɑ-tubulin as an internal control (*n* = 3 technical replicates from six biological replicates for each strain). Osteoclast formation was induced for 4 days. **b** Skeletal preparation of *Rheb1*^*OC*^ or control mice at newborn (P0), aged 1 week (P7) and aged 2 weeks (P14). **c, d** Body weight and length of *Rheb1*^*OC*^ mice aged 2 weeks (body weight *n* = 12 for control, *n* = 11 for *Rheb1*^*OC*^, body length *n* = 9 for control, *n* = 8 for *Rheb1*^*OC*^) and 6 weeks (*n*=6) compared to littermate controls. Unpaired *t* test, *P < 0.05, ****P < 0.000 1. **e** Immunofluorescence staining images of type I or type II collagen deposition in 2-week-old *Rheb1*^*OC*^ mice compared to littermate controls (scale bar, 100 µm and 50 µm). **f** Von Kossa staining images of calcified bone matrix in 2-week-old *Rheb1*^*OC*^ mice compared to littermate controls (scale bar, 100 µm and 50 µm). **g, h** Serum CTX-I levels (*n* = 5 for control, *n* = 6 for *Rheb1*^*OC*^) and P1NP (*n* = 12 for control, *n* = 11 for *Rheb1*^*OC*^) in 2-week-old *Rheb1*^*OC*^ mice compared to littermate controls. Unpaired *t* test, *P < 0.05; n.s., no significance difference. **i** Toluidine blue staining of bone sections from 7 or 14 days after the on-set in drill-hole modeled mice. (*n* = 3 technical replicates from five biological replicates for each strain. Scale bar, 50 µm. Callus: black dashed line). **j–l** ELISA detection analysis of CTX-I (*n* = 9 for control, *n* = 10 for *Rheb1*^*OC*^), TRAP5b (*n* = 9 for control, *n* = 9 for *Rheb1*^*OC*^) or MMP-9 (*n* = 15 for control, *n* = 12 for *Rheb1*^*OC*^) levels in the supernatants from Rheb1-deficient or control osteoclasts cultured on bone slices. Multinucleated osteoclasts were maintained in fresh medium for 2 days before sample collection. Unpaired *t* test, *P < 0.05, **P < 0.01; n.s., no significance difference. All data are presented as mean ± SEM

Given the critical role of Rheb1 in nutrient sensing, we generated osteoclast Rheb1 KO mice (*Rheb1*^*OC*^) (Fig. 1a) to investigate its role in osteoclast function. Analysis of skeletal preparations showed that Rheb1 deletion led to suppressed growth during the postnatal periods prior to weaning (Fig. 1b), with significant reductions in both body weight and length at 2 and 6 weeks of age (Fig. 1c, d). Toluidine blue staining of the growth plate revealed a narrowed height in *Rheb1*^*OC*^ mice (Fig. S1a, c), indicating that osteoclast Rheb1 is crucial for bone development before weaning.

To understand the mechanism behind the growth restriction observed in *Rheb1*^*OC*^ mice, we first examined the in vivo number of osteoclasts. TRAP staining of the metaphyseal spongy bone in 2-week-old mice revealed no significant difference in mature osteoclast numbers between *Rheb1*^*OC*^ and control animals (Fig. S1b, d). However, osteoclast function is required for degrading the cartilaginous matrix, leading to the formation of cartilaginous trabeculae and the subsequent deposition of calcified bone matrix. This process is essential for longitudinal bone growth.^2^ We compared the bony and cartilage trabecular networks between *Rheb1*^*OC*^ and control mice. Type I and type II collagens, major components of the bone extracellular matrix,^37^ showed increased deposition in 2-week-old *Rheb1*^*OC*^ mice (Fig. 1e and Fig. S1e, f). Von Kossa staining also indicated increased bone mineralization in *Rheb1*^*OC*^ mice (Fig. 1f and Fig. S1g). Notably, CTX-I levels, a marker of osteoclast activity in degrading type I collagen, were significantly reduced in *Rheb1*^*OC*^ mice (Fig. 1g), while the serum marker of bone formation, procollagen type 1 N-terminal pro-peptide (P1NP), remained unaffected (Fig. 1h). These results suggest dysregulated extracellular matrix decomposition rather than synthesis, leading to altered bone matrix composition in *Rheb1*^*OC*^ mice. Consistently, bone callus removal was impaired, and bone defect healing was delayed in *Rheb1*^*OC*^ mice (Fig. 1i).

To further elucidate the impact of Rheb1 on osteoclast function, we measured the levels of various resorption degradation products released by osteoclasts. A significant decrease in CTX-I, a fragment released by CTSK cleavage, was detected in the culture supernatants of *Rheb1*^*OC*^ osteoclasts cultured on bone slices (Fig. 1j). In contrast, the level of TRAP5b,^38^ a product indicative of tartrate-resistant acid phosphatase (TRAP) activity, remained comparable to that of control osteoclasts (Fig. 1k). Additionally, the level and activity of matrix metalloproteinase-9 (MMP-9), a type IV collagenase highly expressed in osteoclasts and crucial for cartilage degradation,^39^ were unchanged in Rheb1-deleted osteoclasts (Fig. 1l and Fig. S1h). These results highlight the specific role of Rheb1 in regulating osteoclast function and its importance in the degradation of bone extracellular matrix components.

### Rheb1 deletion impairs osteoclast function via suppressing CTSK production in an mTORC1-independent manner

**Fig. 2:**
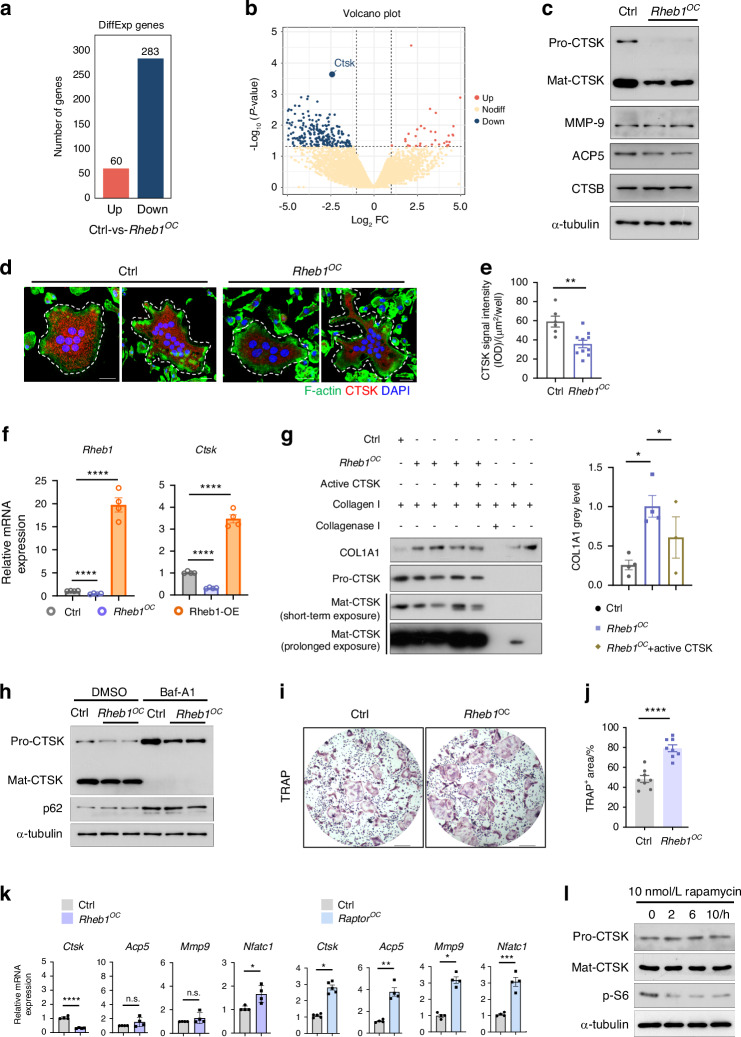
Rheb1 deletion impairs osteoclast function via suppressing CTSK production in an mTORC1-independent manner. **a** Number of differentially expressed genes (DEGs) in the indicated osteoclasts in RNA-seq data. **b** Volcano plot of RNA-seq data to show CTSK is a significantly down-regulated gene in Rheb1-deficient osteoclasts compared to controls (*n* = 3). **c** Immunoblot analysis of CTSK, MMP-9, ACP5 and CTSB protein levels in Rheb1-deficient or control osteoclasts. Approximately 40 kD indicates the pre-proenzyme form, while approximately 35 kDa indicates the active CTSK form. Cells were collected at times when a large majority of multinuclear cells had formed (*n* = 3 technical replicates from more than three biological replicates for each strain). **d, e** Immunofluorescence staining and analysis of the CTSK level in multinucleated Rheb1-deficient or control osteoclasts (scale bar, 20 µm, *n* = 5 for control, *n* = 10 for *Rheb1*^*OC*^). Unpaired *t* test. **P < 0.01. **f** Q-PCR analysis of CTSK and Rheb1 relative mRNA expression in Rheb1-deficient or Rheb1-overexpressing (Rheb1-OE) osteoclasts, compared to the negative controls, respectively (*n* = 4). **g** Immunoblotting analysis to detect type I collagen (0.4 mg/mL) ex vivo degradation in cellular contents from Rheb1-deficient or control osteoclasts. Active CTSK (4E-04 mg/mL) or type I collagenase (1.2 mg/mL) administration as a positive control (*n* = 3 for control, *n* = 4 for *Rheb1*^*OC*^, *n* = 3 for *Rheb1*^*OC*^ + active CTSK). One-way ANOVA. *P < 0.05. **h** Immunoblots of the levels of the pre-proenzyme and active CTSK forms in Rheb1-deficient or control osteoclasts, with or without lysosome inhibitor bafilomycin A1 (10 ng/mL) treatment (*n* = 3 technical replicates from more than three biological replicates). **i–j** Staining for TRAP and analysis of ex vivo osteoclast differentiation in *Rheb1*^*OC*^ or littermate control mice (scale bar, 250 µm); osteoclast differentiation was induced by RANKL and MCSF for 4 days (*n* = 8). **k** Q-PCR analysis to determine relative CTSK, ACP5, MMP-9 and Nfatc1 mRNA expression levels in Rheb1-deficient or Raptor-deficient osteoclasts compared to their controls (*n* = 4). Unpaired *t* test. *P < 0.05, **P < 0.01, ***P < 0.001, ****P < 0.000 1; n.s., no significant difference. **l** Immunoblotting for the protein levels of the CTSK pre-proenzyme and active forms in osteoclasts which were treated with 10 nmol/L rapamycin for 0, 2, 6 or 10 h. A decreased phosphorylated-S6 level indicates mTORC1 inhibition. All data are presented as mean ± SEM

The decreased CTX-I level indicates a potential defect in osteoclast resorption function. To elucidate Rheb1’s role in osteoclast resorption, we performed RNA sequencing on mature osteoclasts generated from both *Rheb1*^*OC*^ and control mice bone marrow-derived monocytes/macrophages (BMDMs). Our analysis revealed that CTSK, a key enzyme for collagen degradation in osteoclasts,^19^ was significantly downregulated in Rheb1-deficient multinucleated osteoclasts (Fig. 2a, b). Further examination showed that CTSK protein levels, including both the proenzyme and mature forms,^40^ were markedly reduced in Rheb1-deficient osteoclasts cultured ex vivo (Fig. 2c–e). In contrast, the levels of other resorption enzymes, such as Tartrate-resistant acid phosphatase type 5 (ACP5), MMP-9 and Cathepsin B (CTSB), remained unaffected. This finding aligns with the observed significant reduction of CTSK in osteoclasts on cancellous bone surfaces in *Rheb1*^*OC*^ mice (Fig. S2a–c). Consistently, increased CTSK mRNA levels were detected in Rheb1-overexpressing osteoclasts (Fig. 2f). To confirm that reduced CTSK production underlies the defective osteoclast function, we incubated cellular contents from control and Rheb1-deficient osteoclasts with type I collagen ex vivo. As anticipated, when compared with the control group, Rheb1-deficient osteoclasts exhibited an impaired ability to degrade type I collagen under equivalent collagen concentrations. Supplementation with active murine CTSK enzyme partially rescued the degradation defect observed with Rheb1 deletion (Fig. 2g). Given that procathepsin K processing depends on lysosome activity^41,42^ and mTORC1 is known to regulate lysosome function,^43^ we found a substantial reduction in procathepsin K in Rheb1-deficient osteoclasts with inhibited lysosome biogenesis (Fig. 2h), suggesting that reduced procathepsin K level is not due to impaired CTSK processing. Additionally, CTSK has a short half-life (~60 min) in both Rheb1-deficient and control osteoclasts (Fig. S2d), indicating that Rheb1 loss does not affect CTSK protein degradation. These findings collectively suggest that Rheb1 specifically regulates procathepsin K production in osteoclasts.

Interestingly, ex vivo assays revealed that osteoclast formation was enhanced in the absence of Rheb1 (Fig. 2i, j), similar to observation in mTORC1-inhibited osteoclasts,^44,45^ suggesting that Rheb1 may have a role in mTORC1-dependent regulation of osteoclast differentiation. However, upon analyzing the expression levels of several osteoclast-specific genes, we found that CTSK mRNA levels were significantly decreased in Rheb1-deficient osteoclasts, while the expression levels of ACP5 and MMP-9 remained unaffected (Fig. 2k). Furthermore, the increased nuclear factor of activated T-cells, cytoplasmic 1 (Nfatc1) level was consistent with the enhanced osteoclast formation observed in Rheb1-deficient osteoclasts. In contrast, the expression levels of these genes were increased in Raptor-deficient osteoclasts (Fig. 2k). These findings suggest that insufficient CTSK might be a marker of osteoclast function defects rather than a determinant of differentiation suppression. Consistent with this, pharmacological inhibition of mTORC1 using the allosteric inhibitor rapamycin significantly reduced the phosphorylation of ribosomal protein S6, a downstream substrate and major effector of mTORC1,^46^ but did not alter CTSK proenzyme and mature form levels in osteoclasts (Fig. 2l). Taken together, these data demonstrate a crucial mTORC1-independent role of Rheb1 in regulating CTSK.

### Rheb1 deletion mainly impairs osteoclast mitochondrial respiration

Notably, our previous findings demonstrated that Raptor-deficient osteoclasts caused osteoporosis in adult mice.^47^ In contrast, micro-computed tomography (micro-CT) analysis of adult *Rheb1*^*OC*^ mice showed a significant reduction in bone mineral content (BMC) without affecting bone microstructure parameters compared to littermate controls (Fig. S3a–c). This discrepancy suggests a specific role for Rheb1 in osteoclast function. Recent studies indicate that Rheb1 localizes in the mitochondrial matrix or outer membrane, regulating mitochondrial respiration.^48–50^ We propose that the metabolic state of osteoclasts likely determine their distinct resorption behaviors. Gene set enrichment analysis (GSEA) revealed that OxPhos is one of the most significantly down-regulated pathways in Rheb1-deficient osteoclasts (Fig. S4a, b). In contrast, glycolysis is inhibited in Raptor-deficient osteoclasts, while OxPhos remains largely unaffected (Fig. S4c, d). These findings imply a mTORC1-independent regulatory role for Rheb1 in mitochondrial function.

**Fig. 3:**
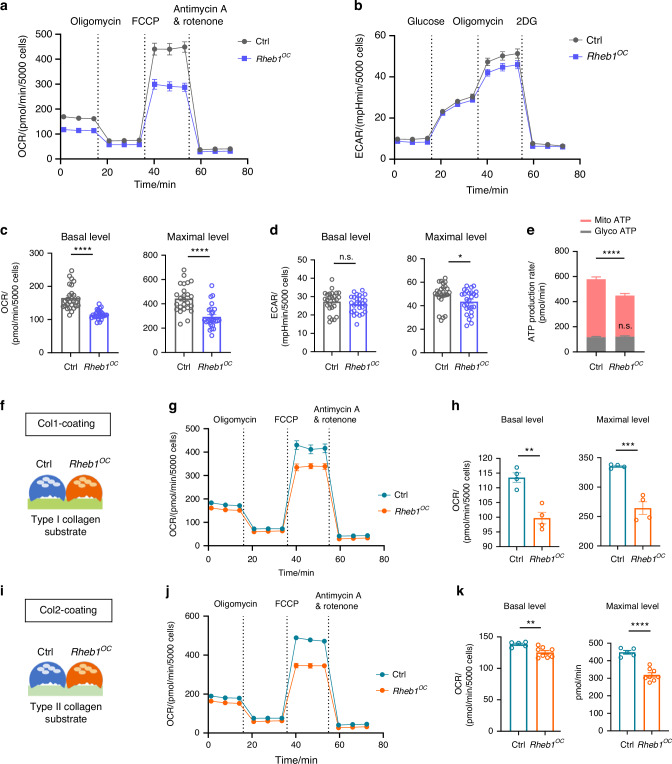
Rheb1 deletion mainly impairs osteoclast mitochondrial respiration. **a** Mitochondrial OCR in the routine, noncoupled (oligomycin), and maximal (FCCP) steady state in Rheb1-deficient osteoclasts or control cells were determined by the real-time Seahorse assay. **b** Extracellular acidification rate (ECAR) in Rheb1-deficient osteoclasts or their respective control cells was determined by the real-time Seahorse assay. **c** The basal respiration and FCCP-induced maximal respiration in the OCR shown in **a** were compared between Rheb1-deficient osteoclasts and their controls (*n* = 26). Unpaired *t* test. ****P < 0.000 1. **d** The basal and maximal glycolysis levels in the ECAR shown in **b** were compared between Rheb1-deficient osteoclasts and their controls (*n* = 26). Unpaired *t* test. *P < 0.05; n.s., no significant difference. **e** The mitochondrial and glycolytic ATP production rates were compared between Rheb1-deficient osteoclasts and their respective control cells (*n* = 26). Unpaired *t* test. ****P < 0.000 1; n.s., no significant difference. **f** Schematic of the type I collagen**-**based ex vivo model. **g** OCR in Rheb1-deficient osteoclasts adherent on type I collagen-coated substrates was determined by the Seahorse assay in real time, which was compared to control cells. **h** The basal and maximal respiration in the OCR shown in **g** (*n* = 4). **i** Schematic of the type II collagen-based ex vivo model. Unpaired *t* test. **P < 0.01, ***P< 0.001. **j** OCR in Rheb1-deficient osteoclast adherent on type II collagen-coated substrates was determined by the Seahorse assay in real time, which was compared to control cells. **k** The basal and maximal respiration in the OCR shown in **j** (*n* = 5 for control, *n* = 8 for *Rheb1*^*OC*^). Unpaired *t* test. **P < 0.01, ****P < 0.000 1. All data are presented as mean ± SEM

To confirm Rheb1’s metabolic role in osteoclast function, we measured energy metabolic pathways in Rheb1-deficient osteoclasts using the Seahorse XF24 Extracellular Flux analyzer. Rheb1-deficient osteoclasts showed a significantly reduced oxygen consumption rate (OCR) (Fig. 3a, c), indicating suppressed mitochondrial respiration. However, the basal extracellular acidification rate (ECAR), an indicator of aerobic glycolysis, was unaffected (Fig. 3b, d). The reduced maximal ECAR level suggests potential involvement of mTORC1.^51,52^ Consistently, mitochondrial ATP production rate was significantly decreased, while glycolytic ATP production rate remained similar to controls, resulting in a reduced total ATP production rate (Fig. 3e). Despite the inhibition of mitochondrial respiration, Rheb1 deletion did not induce oxidative stress (Fig. S5a) or significantly elevate mitophagy (Fig. S5b, c) in osteoclasts.

During endochondral ossification, type I collagen is associated with bone matrix production, while type II collagen is associated with cartilage formation.^53^ Since approximately 90% of the organic matrix of bone is composed of collagens,^19^ we investigated whether Rheb1 modulates the metabolic status of osteoclasts adherent to type I (Fig. 3f) or type II collagen (Fig. 3i). We found that both OCR (Fig. 3g, h) and ECAR (Fig. S5d, e) were suppressed in Rheb1-deficient osteoclasts adherent to type I collagen compared to controls. In contrast, while OCR was also suppressed in Rheb1-deficient osteoclasts on type II collagen (Fig. 3j, k), ECAR remained unaffected (Fig. S5f, g). The varying ECAR levels in Rheb1-deficient osteoclasts adherent to different collagen types suggest distinct underlying mechanisms. However, these findings indicate a specific regulatory role for Rheb1 in osteoclast mitochondrial respiration and underscore the importance of mitochondrial function in osteoclast behavior.

### Mitochondrial respiration is critical to osteoclast procathepsin K production

**Fig. 4:**
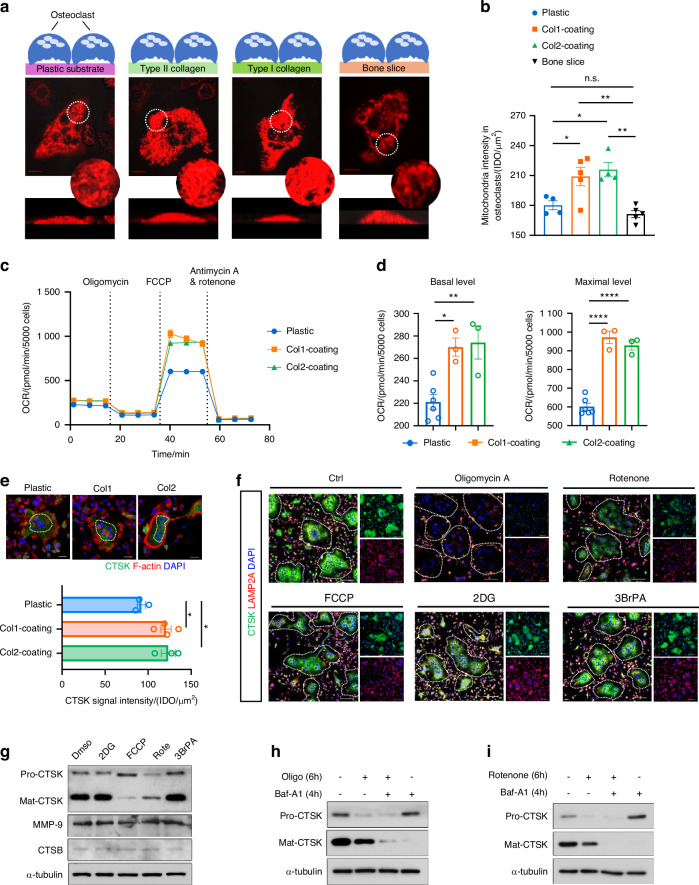
Mitochondrial respiration is critical to osteoclast procathepsin K production. **a** Labeling of mitochondria with Mito Tracker Dyes in osteoclasts adherent on type I collagen-coated or type II collagen-coated interfaces, bone slices or plastic interfaces without collagen-coating, respectively (scale bar, 10 µm). **b** Statistical analysis of immunofluorescence signal density in **a** (*n* = 5 for type I collagen-coating, *n* = 4 for type II collagen-coating, *n* = 5 for bone slice, *n* = 4 for plastic). One-way ANOVA. *P < 0.05, **P < 0.01; n.s., no significant difference. **c** OCR in osteoclasts adherent on type I or type II collagen-coated substrates compared to cells culture on plastic substrates were determined by the Seahorse assay in real-time. **d** The basal respiration and FCCP-induced maximal respiration in the OCR shown in **c** were compared between osteoclasts adherent on collagen-coated substrates and plastic substrates (*n* = 3 for collagen-coating, *n* = 6 for plastic). One-way ANOVA. *P < 0.05, **P < 0.01, ****P < 0.000 1. **e** Immunofluorescence staining analysis to show CTSK protein abundance in osteoclasts adherent on different substrates (scale bar, 100 Pixel, *n* = 3 for plastic, *n* = 4 for type I collagen-coating, *n* = 3 for type II collagen-coating). One-way ANOVA. *P < 0.05. **f** Immunofluorescence staining to show the CTSK expression level in osteoclasts that were treated with oligomycin A (10 nmol/L), rotenone (100 nmol/L), FCCP (1 μmol/L), 2DG (1 μmol/L) or 3BrPA (1 μmol/L) for 6 h (scale bar, 100 µm). **g** Immunoblots of the pre-proenzyme and active CTSK levels in osteoclasts treated with 2DG (1 μmol/L), FCCP (1 μmol/L), rotenone (100 nmol/L) or 3-BrPA (1 μmol/L) for 10 h. The MMP-9 and CTSB expression levels were also detected. DMSO-treated osteoclasts were used as controls. **h, i** Immunoblots of the levels of the CTSK pre-proenzyme and active forms in osteoclasts treated with 10 nmol/L oligomycin A or 100 nmol/L rotenone, with or without lysosome inhibitor bafilomycin A1 (10 ng/mL) treatment for 4 h. Bafilomycin A1 administration was performed followed 2 h later by treatment with OxPhos inhibitors. All data are presented as mean ± SEM

Given that Rheb1 deletion leads to metabolic changes and reduced CTSK in osteoclasts, and that CTSK degrades both type I and type II collagen,^54^ we aimed to determine whether the osteoclast metabolic state depends on the matrix composition. To evaluate whether the osteoclast metabolic state is influenced by the adherence substrate, we established ex vivo models with osteoclasts grown on collagen-coating substrates or bone slices. Fluorescence imaging with Mito-Tracker showed a significant increase in mitochondrial content in mature osteoclasts grown on type I or type II collagen matrices, compared to those grown on bone slices or plastic plates (Fig. 4a, b), suggesting enhanced mitochondrial respiration to support osteoclast activity on collagen-rich matrices. Next, we investigated the metabolic changes in osteoclasts adhering to different substrates. Both basal and maximal OCRs were significantly enhanced in osteoclasts grown on collagen I- or II-coated substrates compared to plastic plates (Fig. 4c, d). Basal and maximal ECARs were unchanged in osteoclasts on collagen I but increased in those on collagen II (Fig. S6a, b), suggesting collagen augments both OCR and ECAR. Notably, when osteoclasts adhered to type I or type II collagen, CTSK protein levels in the perinuclear area increased in response to fresh culture medium (Fig. 4e), indicating a heightened requirement for newly synthesized CTSK proenzyme in osteoclasts on collagen-containing substrates.

Glycolysis is generally considered the preferred energetic pathway for bone resorption in osteoclasts,^11^ while mitochondrial OxPhos is the main energy source for osteoclast differentiation.^55^ To determine the metabolic pathway supporting CTSK production, we measured CTSK levels in osteoclasts treated with glycolysis or mitochondrial stress components (2-deoxy-D-glucose (2DG), 3-bromopyruvate (3BrPA), carbonyl cyanide 4-(trifluoromethoxy) phenylhydrazone (FCCP), rotenone and oligomycin A). Surprisingly, CTSK expression levels were not significantly affected by 3BrPA (Fig. S6c) or 2DG treatment (Fig. S6d) but were profoundly reduced by rotenone or oligomycin A (Fig. 4f and Fig. S6e, f), suggesting that glycolysis is not the preferred bioenergetics pathway for CTSK production. Consistently, mitochondrial inhibitor reduced CTSK levels in osteoclasts attached to collagen I or II substrates (Fig. S6g, h). Notably, rotenone treatment specifically reduced CTSK levels without affecting other resorption enzymes like MMP-9 and CTSB (Fig. 4g), indicating specific regulation by mitochondrial respiration. Oligomycin A (Fig. S6i) or rotenone (Fig. S6j) treatment significantly reduced both procathepsin K and active CTSK levels in a time-dependent manner, whereas FCCP likely suppressed CTSK processing without similar reductions (Fig. 4g). The different effects of FCCP and oligomycin/rotenone suggest that mitochondrial uncoupling is not crucial for procathepsin K production. Consistent with findings in Rheb1-deficient osteoclasts, oligomycin or rotenone treatment suppressed CTSK proenzyme production (Fig. 4h, i). These ex vivo data indicate that osteoclasts have a particular preference for the mitochondrial metabolic pathway when producing CTSK.

### Acetyl-CoA availability is essential in supporting osteoclastic CTSK production

**Fig. 5:**
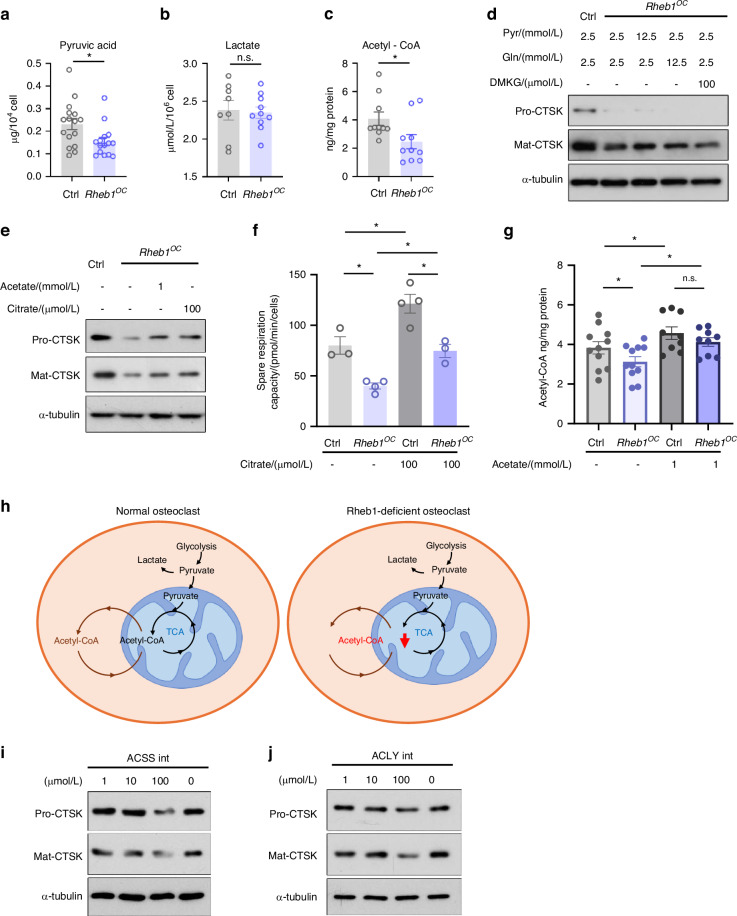
Acetyl-CoA availability is essential in supporting osteoclastic CTSK production. a Intracellular pyruvate level was determined and analyzed in Rheb1-deficient osteoclasts compared to controls (*n* = 16 for control, *n* = 15 for *Rheb1*^*OC*^). Unpaired *t* test. *P < 0.05. **b** Intracellular lactate level was determined and analyzed in Rheb1-deficient osteoclasts compared to controls (*n* = 8 for control, *n* = 10 for *Rheb1*^*OC*^). Unpaired *t* test, n.s., no significant difference. **c** Intracellular acetyl-CoA level was measured and analyzed in Rheb1-deficient osteoclasts compared to controls (*n* = 10). Unpaired *t* test. *P < 0.05. **d** Immunoblots of the pre-proenzyme and active CTSK protein levels in Rheb1-deficient or respective control osteoclasts grown in media containing additional 10 mmol/L pyruvate, 10 mmol/L glutamine or 100 μmol/L DMKG for 6 h, respectively. **e** Immunoblots of pre-proenzyme and active CTSK protein levels in Rheb1-deficient or respective control osteoclasts incubated with 1 mmol/L acetate or 100 μmol/L citrate for 6 h, respectively. **f** The spare respiration capacity was compared between Rheb1-deficient osteoclasts and their controls, with or without citrate incubation for 6 h (*n* = 3 or 4). One-way ANOVA. *P < 0.05. **g** Intracellular acetyl-CoA levels were measured and analyzed in Rheb1-deficient osteoclasts compared to controls, with or without 1 mmol/L acetate incubation for 6 h (*n* = 11). One-way ANOVA. *P < 0.05, n.s., no significant difference. **h** Schematic of acetyl-CoA availability defect in Rheb1-deficient osteoclasts. **i** Immunoblots of the pre-proenzyme and active CTSK protein levels in osteoclasts incubated with or without different doses of ACCS2 inhibitor for 6 h. **j** Immunoblots of the pre-proenzyme and active CTSK protein levels in osteoclasts incubated with or without different doses of ACLY inhibitor SB 204990 for 6 h. All data are presented as mean ± SEM

Rotenone and oligomycin both reduce the OCR and metabolite availability,^56,57^ while FCCP acts as a potent uncoupler of mitochondrial OxPhos, thereby elevating the OCR.^58^ The inhibition of CTSK production under mitochondrial stress implies that metabolites generated from mitochondrial respiration are crucial for osteoclast CTSK production. Examination of central metabolites revealed a decreased pyruvate content in Rheb1-deficient osteoclasts (Fig. 5a), while lactate levels remained similar to controls (Fig. 5b). These results suggest that the glycolysis-tricarboxylic acid cycle (TCA) cycle flux is attenuated to a certain extent in Rheb1-deficient osteoclasts. A significantly lower level of acetyl-CoA was detected in Rheb1-deficient osteoclasts compared to controls (Fig. 5c), indicating down-regulation of acetyl-CoA generation due to Rheb1 deficiency. Given that pyruvate and glutamine are two major carbon sources entering the TCA cycle, we supplemented Rheb1-deficient osteoclasts with pyruvic acid or glutamine to identify what stimulates CTSK production. To exclude nutrient effects on osteoclast differentiation, pyruvate or glutamine was supplied after multinuclear osteoclasts had formed (Fig. S7a). Both pyruvate (Fig. S7b) and glutamine (Fig. S7c) significantly elevated the expression of proenzyme and active CTSK in multinucleated osteoclasts. However, these treatments did not restore CTSK level in Rheb1-deficient osteoclasts when compared to controls (Fig. 5d). Similarly, the addition of dimethyl-α-ketoglutarate (DMKG), a key intermediate in glutamine metabolism, failed in restoring CTSK levels in Rheb1-deficient osteoclasts (Fig. 5d and Fig. S7d). Interestingly, the addition of acetate (Fig. S7e, f) or citrate (Fig. S7g, h) increased CTSK protein levels, but not MMP-9 or ACP5, in mature osteoclasts. Administering acetate or citrate to Rheb1-deficient multinucleated osteoclasts increased both procathepisn K and mature CTSK protein levels (Fig. 5e). Given that acetate and citrate can be converted to acetyl-CoA, incubation with citrate restored the spare respiration capacity (Fig. 5f), while acetate restored cellular acetyl-CoA levels in Rheb1-deficient osteoclast (Fig. 5g). The transcriptional regulation of enzymes involved in acetyl-CoA conversion remained unaffected by Rheb1 deletion (Fig. S7i). These findings indicate that mitochondrial malfunction resulting from Rheb1 deletion primarily impairs acetyl-CoA availability (Fig. 5h). To validate the centrality of acetyl-CoA availability for CTSK production, we examined CTSK levels in osteoclasts incubated with acetyl-CoA synthase 2 (ACCS2) or ATP citrate lyase (ACLY) inhibitors. Notably, administration of ACCS2 inhibitor reduced both procathepsin K and mature CTSK production (Fig. 5i). Similarly, ACLY inhibitor SB 204990 also decreased CTSK levels (Fig. 5j). These findings indicate that mitochondrial respiration is essential for CTSK expression in osteoclasts, potentially through providing acetyl-CoA via the TCA cycle.

Acetyl-CoA availability influences histone acetylation, thereby regulating transcription.^59^ However, high concentrations of acetate significantly reduced both CTSK mRNA and protein levels in osteoclasts (Fig. S8a, b), while increasing global histone H3 acetylation (Fig. S8c). These data align previous studies showing that global acetylation suppresses osteoclast formation.^60^ Given the overall reduction in acetyl-CoA levels in Rheb1-deficient osteoclasts and the restoration of CTSK protein levels with specific acetate concentrations, it is likely that the insufficient CTSK levels in Rheb1-deficient osteoclasts are due to a decreased nucleocytosolic pool of acetyl-CoA.

Mitochondrial respiration suppression in Rheb1-deficient osteoclasts might also be due to decreased levels of NAD-dependent protein deacetylase sirtuin-3 (SirT3), which enhances mitochondrial enzyme activity.^61–63^ Consistent with our hypothesis, mitochondrial SirT3 levels decreased in both Rheb1-deficient osteoclasts (Fig. S9a) and tumor necrosis factor ligand superfamily member 11 (RANKL)-incubated Rheb1 KO Raw264.7 cells (Fig. S9b, c), with additional acetate partially reversing this reduction. However, SirT3 levels were not reduced in Rheb1 KO Raw264.7 cells without RANKL stimulation (Fig. S9d), indicating a specific regulation by Rheb1 on SirT3 in osteoclasts, distinct from their precursors. These results suggest that acetate helps maintain osteoclast mitochondrial activity when Rheb1 is absent.

In conclusion, our data suggest that acetyl-CoA availability is crucial for procathepisn K production in osteoclasts and is regulated by Rheb1. The ability of osteoclasts to generate acetyl-CoA from internal or external sources is vital for maintaining CTSK levels.

### Ethanol intake causes a poor fracture healing due to increased CTSK production

**Fig. 6:**
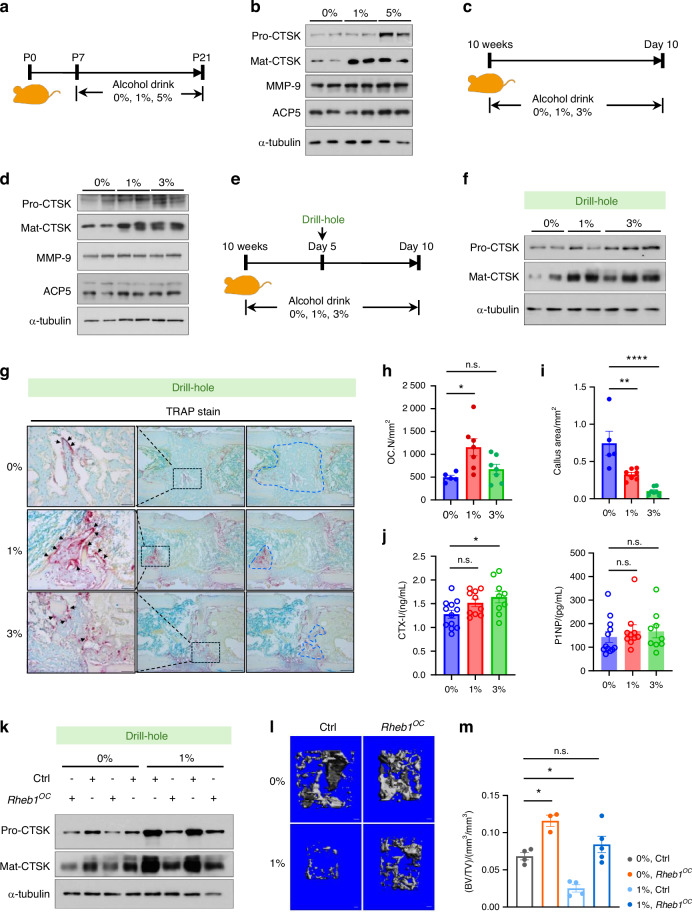
Ethanol intake causes a poor fracture healing due to increased CTSK production. **a** A schematic pattern of a mouse alcohol consumption model during development. **b** Immunoblots of CTSK, MMP-9 and ACP5 protein levels in lysates prepared from P21 mice epiphyseal cancellous bone tissues, with ɑ-tubulin as an internal control. **c** A schematic pattern of an adult mouse alcohol consumption model. **d** Immunoblotting analysis of CTSK, MMP-9 and ACP5 levels in lysates prepared from epiphyseal cancellous bone tissues of alcohol-consuming mice in **c**, with ɑ-tubulin as an internal control. **e** A schematic pattern of the adult mouse alcohol consumption model with or without a drill-hole bone defect. **f** Immunoblot analysis of CTSK protein levels in drilled hole bone tissues from mice in **e**. **g, h** Analysis of staining for TRAP (black arrow: TRAP-positive cell) to show osteoclastogenesis in mice consuming alcohol following drill-hole surgery (scale bar, left panel 50 µm, right panel 200 µm). One-way ANOVA. *P < 0.05, n.s., no significant difference. **i** Callus size (shown in **g**, blue dashed line) analysis in drill-hole model mice with or without alcohol consumption. One-way ANOVA. **P < 0.01, ****P < 0.000 1. **j** Serum CTX-I and P1NP levels in mice modeling in **e**. One-way ANOVA. *P < 0.05, n.s., no significant difference. **k** Immunoblotting analysis of the CTSK protein level in callus tissue from *Rheb1*^*OC*^ drill-hole mice and their littermate controls. With ɑ-tubulin as an internal control. **l, m** Micro-CT analysis to compare callus size in drill-hole surgery *Rheb1*^*OC*^ mice to littermate controls in the context of alcohol consumption (scale bar, 100 µm). One-way ANOVA. *P < 0.05, n.s., no significant difference. All data are presented as mean ± SEM

Acetate, a byproduct of hepatic alcohol metabolism, elevates circulating acetate levels following alcohol consumption.^64,65^ To investigate whether acetate affects CTSK during endochondral ossification in the developing skeleton, we modeled alcohol consumption with equivalent caloric intake in postnatal day 7 (P7) mice (Fig. 6a). Our observations indicated that intake of 5% and 1% ethanol did not significantly impact body weight (Fig. S10a, b). However, both procathepsin K and its active form were abundantly expressed in the epiphyseal regions of all alcohol-fed mice, while other osteoclast matrix-degrading enzymes were not (Fig. 6b). This occurred despite no changes in serum CTX-I and P1NP levels (Fig. S10c). In adult mice (Fig. 6c), CTSK levels were also specifically upregulated (Fig. 6d). To further confirm the sensitivity of skeletal CTSK production to acetyl-CoA availability in adults, we modeled alcohol consumption in mice with drill-hole bone defects (Fig. 6e). We observed non-healing fractures in mice offered 1% ethanol, with severity increasing at 3% ethanol intake (Fig. S10d), without affecting body weight (Fig. S10e) and inflammatory response (Fig. S10f). Compared to the diet group without alcohol, 1% ethanol intake in mice resulted in increased CTSK production at the fracture site (Fig. 6f), higher osteoclast numbers (Fig. 6g, h), and reduced bone callus (Fig. 6i). In mice with 3% ethanol access, there was no significant increase in osteoclast numbers, but bone callus size significantly decreased. Increased bone callus removal correlated with higher CTSK levels detected by immunoblotting, indicating that abundant CTSK is associated with a high callus removal rate. The increased serum CTX-I levels in alcohol-consuming mice, with P1NP levels similar to controls (Fig. 6j), suggest that alcohol consumption contributes more to bone resorption than to bone formation during bone remodeling. Our observation of increased CTSK production in heart tissue following alcohol intake in mice (Fig. S10g) might further support the regulation of CTSK by Acetyl-CoA availability. Collectively, we concluded that excessive CTSK-mediated resorption is the primary factor interfering with fracture healing in the context of alcohol consumption.

Given that Rheb1 deletion limits CTSK abundance, we investigated whether this deletion could mitigate the effects of alcohol consumption on callus removal. We modeled alcohol consumption in osteoclast Rheb1-deficient mice and littermate controls five days before inducing drilled-hole injury. Alcohol consumption significantly elevated serum ethanol concentrations in both *Rheb1*^*OC*^ and control mice (Fig. S10h), without affecting inflammatory response (Fig. S10i). We found that while CTSK levels increased in alcohol-consuming control mice, they were reduced in *Rheb1*^*OC*^ mice (Fig. 6k), resulting in a deceleration of bone callus removal (Fig. 6l, m). This suggests that Rheb1 deletion confers partial resistance to the adverse effects of alcohol consumption on bone remodeling.

Together, these in vivo data corroborate that acetyl-CoA availability is essential for osteoclast-specific CTSK production. This implies that approaches altering osteoclast acetyl-CoA availability may effectively interfere with CTSK function. Understanding the regulatory mechanisms by which alcohol consumption increases CTSK levels may inform dietary recommendations during fracture healing.

## Discussion

**Fig. 7:**
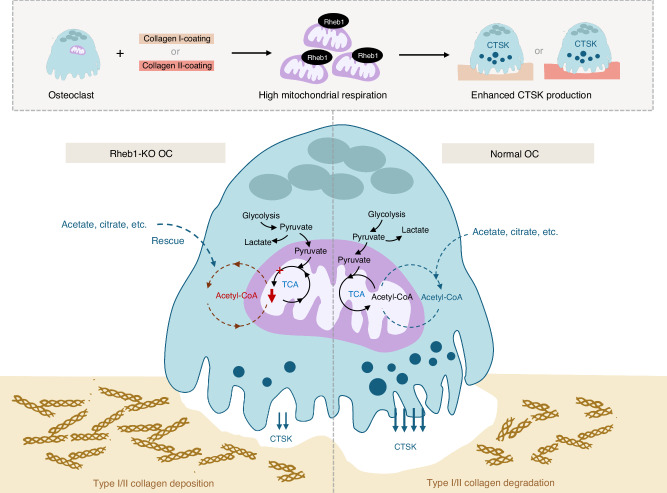
Acetyl-CoA generation is central to CTSK production. Rheb1 localizes to mitochondria and regulates mitochondrial respiration in multinucleated osteoclasts. When osteoclasts attach to substrates rich in type I or type II collagen, mitochondrial respiration is enhanced, promoting CTSK protein expression. Rheb1 deletion impairs mitochondrial respiration in osteoclasts, reduces acetyl-CoA production, and specifically inhibits CTSK expression and the collagen degradation ability of osteoclasts. Under physiological conditions, mitochondria-derived acetyl-CoA is necessary for osteoclasts to produce CTSK, though other sources of acetyl-CoA can also assist in CTSK production. The availability of acetyl-CoA determines the level of CTSK production in osteoclasts, thereby affecting bone and cartilage matrix homeostasis. CTSK cathepsin K, OC osteoclast, TCA tricarboxylic acid cycle

Osteoclast is critical for maintaining bone homeostasis.^2,66^ Actively resorbing osteoclasts are able to dissolve bone mineral with acid, degrade collagen fibers and a wide array of other matrix-residing proteins using various specialized enzymes in a coordinated fashion.^2^ The components of the structural bone matrix vary at different stages of endochondral ossification. Understanding how osteoclasts fine-tune their resorptive activity by relying on various functional enzymes, and the mechanisms regulating this process, could provide new opportunities for treating bone modeling and remodeling disorders. In this study, we uncover a unique role for mitochondria and acetyl-CoA availability in regulating osteoclast function, particularly in collagen degradation (Fig. 7).

The role of mitochondria and energy metabolism in osteoclasts is multifaceted. Several studies have associated mitochondrial OxPhos with osteoclast differentiation^9,12,67^ and glycolysis with bone resorption^11,67^; however, the reason for the abundance of mitochondria in osteoclasts remains unclear. The organization of the cytoskeleton is crucial for osteoclast to form a sealing zone.^68^ Evidence suggests that mitochondria may influence osteoclast function by regulating the cytoskeleton.^69,70^ In our study, we observed that osteoclasts adherent to type II, but not type I collagen, are more likely to form actin rings, suggesting osteoclast recognition of type II collagen may promote cytoskeletal filaments assembly. Additionally, the varying ECAR levels in Rheb1-deficeint osteoclasts under different conditions suggest that glycolysis might be involved in distinct functions of osteoclasts adherent to different collagen types. However, whether there are differences in metabolic regulation of osteoclasts in recognizing these two types of collagens needs more detailed research. Our experimental design and results suggest that this difference may be relevant to the type of matrices but are probably independent of the stiffness of the interface to which the osteoclasts adhere. Given that CTSK abundance and SirT3 levels in multinucleated osteoclasts were not affected by inhibitor of integrin α_v_β_3_ (Fig. S11a–c), we hypothesize that the signaling pathway mediated by integrin engagement with RGD epitopes may not be directly involved in the mitochondrial regulation of CTSK levels, however, further evidence is required.

Under physiological and normoxic conditions, mitochondria are the primary source of acetyl-CoA in mammalian cells, contributing significantly to the cytosolic acetyl-CoA pool.^7,59^ Given its role in numerous biological processes, it is unsurprising that acetyl-CoA availability regulates osteoclast function. Importantly, our findings indicate a specific preference for mitochondria generated acetyl-CoA in regulating CTSK, the most potent collagenase in osteoclasts. CTSK is a short-lived protein; osteoclasts must modulate its abundance in response to stimuli to regulate resorption behavior effectively. Our study demonstrated that optimal doses of pyruvate, glutamine, acetate, or citrate efficiently and specifically stimulate CTSK protein abundance in osteoclasts, indicating that acetyl-CoA utilization is crucial for maintaining CTSK-related functions in bone. Under conditions of mitochondrial dysfunction, cells may produce acetyl-CoA through metabolic circuitries in the cytosol,^59^ providing an alternative mechanism to promote CTSK protein expression.

Chronic alcohol consumption is strongly associated with tissue damage.^65^ Rodent studies have shown that an ethanol-rich diet reduces bone matrix protein expression, inhibits osteogenesis^30,31^ and promotes osteoclastogenesis.^29^ Although studies suggest that alcohol consumption may promote bone loss via inflammation,^30,71^ our findings indicate that even limited alcohol intake, without affecting circulating inflammatory factor levels, significantly increases CTSK abundance in bone and accelerates bone callus removal, suggesting that patients consuming alcohol with fractures might have an increased risk of poor fracture union. Impaired bone callus removal observed in *Rheb1*^*OC*^ mice suggests that insufficient CTSK is also harmful to fracture healing. Our findings provide novel insights into the underlying mechanisms of osteoclast behavior in regulating fracture repair and suggest that optimal CTSK abundance is crucial for bone quality. Furthermore, we found that the effect of alcohol consumption on the CTSK levels in osteoclasts was not reflected by changes in osteoclast numbers, which suggests a potential differential role of various functional enzymes in osteoclasts during bone remodeling and indicates that the regulatory mechanisms of energy metabolism in osteoclasts may be crucial for the differential expression of these functional enzymes.

In summary, our findings indicate that under physiologic conditions, mitochondrial acetyl-CoA generation is essential for maintaining osteoclast CTSK function, whereas increased acetyl-CoA availability provides an alternative means to modulate CTSK. Our data provide novel insights into the metabolic regulation of CTSK production in osteoclasts, emphasizing the pivotal and specific roles of mitochondrial respiration in sustaining CTSK-mediated collagen degradation.

## Materials and methods

### Mice

All mice were maintained at constant temperature (22 °C) and humidity (60%) in a 12-h light/dark cycle according to the guidelines of the Association for Assessment and Accreditation of Laboratory Animal Care. *Ctsk*^Cre^ mice, carrying a nuclear-localized Cre recombinase inserted into the *Ctsk* gene (a generous gift from Prof. Shigeaki Kato, University of Tokyo), were crossed with *Rheb1* floxed mice (a generous gift from Prof. Bo Xiao, Southern University of Science and Technology) to generate *Ctsk*^*Cre*^*Rheb*^*floxp*/*floxp*^ mice in which the osteoclastic lineage exhibited specifically deleted *Rheb*. *Ctsk*^Cre^ mice were crossed with *Raptor* floxed mice (JAX stock #013188, Jackson Laboratory, ME, USA) to generate Ctsk^Cre^Raptor^floxp/floxp^ mice in which the osteoclastic lineage exhibited specifically deleted *Raptor*. C57BL/6 mice were purchased from the Southern Medical University Laboratory Animal Center (Guangzhou, China). All mice were maintained on a C57BL/6 J background and bred under specific-pathogen-free conditions. Food and water were available *ad libitum*. All mice studies were performed with the approval of the Southern Medical University Animal Care and Use Committee in accordance with guidelines for the ethical treatment of animals.

### Osteoclast culture

BMDMs were isolated from femurs and tibias of 6–8-week-old mice and cultured in α-Minimum Essential Medium (α-MEM) (Invitrogen, Cat. No. 12571063, Carlsbad, CA, USA) with 10% fetal bovine serum (FBS). Primary culture experiments were performed in Class II Biological Safety Cabinets, in a certified PC2 laboratory. All cells were maintained in a humidified CO_2_ incubator under 5% CO_2_ at 37 °C. For in vitro differentiation, BMDMs were digested, counted, seeded and cultured in α-MEM (10% FBS) with 20 ng/mL macrophage colony-stimulating factor (M-CSF) (315-02, PeproTech, Cranbury, NJ, USA) and 50 ng/mL RANKL (462-TR-010, R&D system, Minneapolis, MN, USA). Cells were cultured for 3–6 days, with the medium replaced every second day. For assays on a collagen-coated interface, diluted type I collagen (C8062, Solarbio, Beijing, China) or type II collagen (C8000, Solarbio) solution at 20 ng/μL in sterile water was added with diluent to a 24-well plate with glass coverslips, with 500 μL per well, while 65 μL per well of Seahorse XFe96/V3 was added to a microplate. Collagen solution was incubated overnight in a humidified CO_2_ incubator under 5% CO_2_ at 37 °C. Collagen-coating wells in all experiments were washed with α-MEM twice before used. To ensure a standardized extracellular environment and a synchronized cell state, culture medium was replaced before treatment in all experiments. Cells were treated with inhibitors or substrates according to a time or concentration gradient. Inhibitors and substrates used were as follows: rotenone (HY-B1756, MedChemExpress, NJ, USA), oligomycin A (HY-16589, MedChemExpress), FCCP (HY-100410, MedChemExpress), 2-DG (HY-13966, MedChemExpress), 3BrPA (HY-19992, MedChemExpress), bafilomycin A1 (HY-100558, MedChemExpress), pyruvate (103578-100, Agilent Technologies, Santa Clara, CA, USA), glutamine (103579-100, Agilent Technologies), citrate (C0759, Sigma Aldrich, St. Louis, USA), acetate (AM9740, Invitrogen, Waltham, Massachusetts, USA), DMKG (349631, Sigma Aldrich, St. Louis, USA), ACSS2 inhibitor (HY-148104, MedChemExpress), ACLY inhibitor (HY-16450, MedChemExpress) and integrin α_v_β_3_ inhibitor (HY-P0023, MedChemExpress). In some experiments, membrane-impermeant acetyl-CoA (HY-113596, MedChemExpress) was used as a negative control.

### Alcohol diet model

Adult male mice (aged 10 weeks) were randomized and acclimated to the AIN93M liquid diet #TP4020 or control diet #TP4020C. The amount of liquid intake was determined by the volume difference between to the volume offered and the remaining volume. The control mice were fed by using the average milliliters of diet consumed by all of the alcohol-drinking mice. Calories derived from ethanol were estimated as 7.2 kcal/g. For the femur drill-hole model, mice were fed a liquid diet 4 days before bone drill-hole modeling. The alcohol-drinking group started on 1% ethanol on the first day of our studies. For adult C57BL/6 J, the ethanol was increased to 2% on day 2, to 3% on day 3 and continued at 3% for the remainder of the study. Because a diet with 3% ethanol caused a reduced diet intake, the ethanol was maintained at 1% for the transgenic mice used in the study. P7 C57BL/6 J male mice were randomized and acclimated to the AIN93G liquid diet #TP4010 or control diet #TP4010C. The mice were breastfed and fed with the corresponding concentrations of alcohol liquid feed daily through gavage, with 60 μL per volume twice daily. Similarly, the alcohol-drinking group started on 1% ethanol on the first day. For the 5% alcohol-drinking group, the ethanol was increased to 2% on day 2, to 3% on day 3, to 4% on day 4, to 5% on day 5, and continued at 5% for the remainder of the study. Liquid food consumption was recorded daily, and the body weight was recorded every other day.

### Collagenolytic activity assay

Osteoclasts were induced from BMDMs by adding 20 ng/mL M-CSF and 50 ng/mL RANKL. Cells were lysed, and the cellular contents were harvested once a majority of multinucleated cells had formed. For the extracellular collagenolytic activity assay, type I rat collagen (0.4 mg/mL) was incubated with cellular contents, with or without active mouse recombinant CTSK (mCTSK, SRP6558, Sigma-Aldrich,) in the activity buffer as described previously^72^ (100 mmol/L sodium acetate, 2.5 mmol/L 1,4-Dithioerythritol and 2.5 mmol/L Ethylenediaminetetraacetic acid, pH 5.5). Collagen digestion was conducted at 37 °C for 20 h in water bath. Positive controls included 0.4 mg/mL collagen type I and 4E-04 mg/mL mCTSK. Negative controls included 0.4 mg/mL collagen type I without cellular content or with 1.2 mg/mL type I collagenase. Each sample contained equal initial concentrations of collagen and cellular contents, respectively. Following the reaction, samples were subjected to SDS-Polyacrylamide Gel Electrophoresis (SDS-PAGE) using 10% tris/glycine gels, and subsequently analyzed by immunoblotting.

### Supplementary information

Supplemental Information

Supplemental Figure 1

Supplemental Figure 2

Supplemental Figure 3

Supplemental Figure 4

Supplemental Figure 5

Supplemental Figure 6

Supplemental Figure 7

Supplemental Figure 8

Supplemental Figure 9

Supplemental Figure 10

Supplemental Figure 11

## Data Availability

All data needed to evaluate the conclusions in the paper are present in the paper and the Supplementary Materials. Further information and reasonable requests for resources, reagents and other experimental data that support the findings within this paper are available from the Lead Contact, Yue Zhang (yugi@smu.edu.cn, yugiben@163.com).
