# Supplementary material for: Osteoclasts control endochondral ossification via regulating acetyl-CoA availability
Source: Bone Res. 2024 Aug 28;12:49. doi: 10.1038/s41413-024-00360-6 (PMC11358419; doi:10.1038/s41413-024-00360-6)
Supplement: Supplementary file 1 — Supplemental Information [file 41413_2024_360_MOESM1_ESM.docx]

**Supplementary Information for**

**Osteoclasts control endochondral ossification via regulating acetyl-CoA availability**

Dai-Zhao Deng^1^, Xian-Ming Liu^1^, Wen-Lan Huang^1^, Si-Rui Yuan^1^, Gen-Ming Liu^1^, Shan-Shan Ai^3^, Yi-Jie Fu^1^, Hao-Kun Xu^1^, Xin-Yi Zhang^1^, Shi-Hai Li^1^, Song Xu^2^, Xiao-Chun Bai^1^, Yue Zhang^1,4^

^1^Department of Cell Biology, School of Basic Medical Science, Southern Medical University, Guangzhou, 510515, Guangdong, China

^2^Nanfang Hospital, Southern Medical University, Guangzhou, 510515, Guangdong, China

^3^Department of Physiology, School of Basic Medical Science, Southern Medical University, Guangzhou, 510515, Guangdong, China

^4^Lead contact

These authors contributed equally: Dai-Zhao Deng, Xian-Ming Liu, Wen-Lan Huang

Correspondence to: Yue Zhang ([yugi@smu.edu.cn](mailto:yugi@smu.edu.cn), Tel: 86-20-61647584, Fax: 86-20-61648208) or Xiao-Chun Bai ([baixc15@smu.edu.cn](mailto:baixc15@smu.edu.cn)) or Song Xu ([iwbaking@163.com](mailto:iwbaking@163.com))

This file includes:

Supplemental Figure legends 1-11

Table 1

Supplemental Materials and Methods

**Supplemental Figure legends**

**Fig. S1 Global skeletal phenotypes analysis of Rheb1^OC^ mice compared to controls, data related to main Figure 1. a** Growth plate toluidine blue staining of 2-week-old Rheb1^OC^ mice compared to littermate controls (scale bar, 500 µm and 100 µm). **b** Staining for TRAP with overview of epiphysis sections from 2-week-old Rheb1^OC^ mice compared to littermate controls (scale bar, 200 µm). **c** Growth plate size analysis of 2-week-old Rheb1^OC^ mice compared to littermate controls (n = 7 for control, n = 6 for Rheb1^OC^). Unpaired t test. ****p* < 0.001. **d** Analysis of epiphyseal osteoclast number by staining for TRAP in 2-week-old Rheb1^OC^ mice compared to littermate controls (n = 8 for control, n = 9 for Rheb1^OC^). Unpaired t test, n.s., no significant difference. **e, f** Immunofluorescence staining analysis of type I (n = 6 for control, n = 7 for Rheb1^OC^) or type II (n = 4 for control, n = 3 for Rheb1^OC^) collagen deposition in 2-week-old Rheb1^OC^ mice compared to littermate controls. Unpaired t test. **p* < 0.05, ****p* < 0.001. **g** Von Kossa staining analysis of calcified bone matrix in 2-week-old Rheb1^OC^ mice compared to littermate controls (n = 3 for control, n = 3 for Rheb1^OC^). Unpaired t test. ***p* < 0.01. **h** Gelatinase spectrum analysis of MMP-9 activity in Rheb1-deficient osteoclasts compared to controls. (n = 3 technical replicates from three biological replicates for each strain). All data are presented as mean ± SEM.

**Fig. S2 Rheb1 deletion suppresses CTSK expression and impairs osteoclast function, data related to main Figure 2. a** Immunofluorescence staining image to show the decreased CTSK level in osteoclasts from Rheb1^OC^ mice compared to littermate controls attached on the surface of bone. MMP-9 signal indicates osteoclast location (scale bar, 5 µm). **b–c** Immunofluorescence staining analysis of CTSK or MMP-9 signal intensity in (d) (n = 3 for control, n = 4 for Rheb1^OC^). Unpaired t test. **p* < 0.05, n.s., no significant difference. **d** CTSK protein life measured in Rheb1-deficient osteoclasts or respective controls at 0, 30, 60, and 90 minutes of treatment with 500 ng/mL CHX, with ɑ-tubulin as an internal control (n = 2 technical replicates from three biological replicates). All data are presented as mean ± SEM.

**Fig. S3 Micro-CT analysis of long bones in Rheb1^OC^ and littermate control mice.**

**a–b** 3D (scale bar, 1 mm or 100 µm) and 2D (scale bar, 1 mm) micro-CT reconstruction of distal femoral bone in 10–12 week-old Rheb1^OC^ mice or littermate controls. **c** Bone parameters in micro-CT analysis of Rheb1^OC^ mice femoral bone compared to their littermate controls (n = 6 for control, n = 6 for Rheb1^OC^). Unpaired t test. **p* < 0.05, n.s., no significant difference. All data are presented as mean ± SEM.

**Fig. S4 OxPhos is down-regulated in Rheb1-deficient but not in Raptor-deficient osteoclasts, data related to main Figure 3. a** OxPhos heat map showing the significant genes expressed in the indicated osteoclasts in the RNA-seq data from mice (n = 3). **b** Enrichment plot of RNA-seq analysis of Rheb1-deficient osteoclasts compared to controls on the OxPhos gene set from the gene set enrichment analysis (GSEA). **c-d** GSEA was performed with the glycolysis gene sets (c) and OxPhos gene sets (d) in Raptor-deficient osteoclasts compared to controls. NES, normalized enrichment score.

**Fig. S5 Effects of Rheb1 deletion on mitophagy, ROS stress and glycolysis in osteoclasts, data related to main Figure 3. a** Cellular ROS assay was performed to determine the ROS level in osteoclasts. Relative fluorescence intensity shown to compare between Rheb1-deficient osteoclasts and control cells (n = 4). Unpaired t test, n.s., no significant difference. **b** Co-labeling mitochondria and lysosome with Mito Tracker Dyes and Lyso Tracker Dyes in Rheb1-deficient osteoclasts or their respective controls (scale bar, 50 µm). **c** Scatterplot of red and green pixel intensities of osteoclasts following incubation with Mito Tracker Dyes and Lyso Tracker Dyes (left panel); Pearson's correlation coefficients of images of internalized red-mito and green-lyso in osteoclasts (n = 3). Unpaired t test, n.s., no significant difference. **d** ECAR in Rheb1-deficient osteoclasts or their respective control cells adherent on type I collagen-coated substrates were determined by the real-time Seahorse assay. **e** The basal and maximal glycolysis levels in the ECAR shown in (d) (n = 4). Unpaired t test. ***p* < 0.01, ****p* < 0.001. **f** ECAR in Rheb1-deficient osteoclasts or their respective control cells adherent on type II collagen-coated substrates were determined by the real-time Seahorse assay. **g** The basal (n = 5) and maximal (n = 5) glycolysis levels in the ECAR shown in (f). Unpaired t test, n.s., no significant difference. All data are presented as mean ± SEM.

**Fig. S6 Mitochondrial respiration is critical to osteoclasts adherent on collagen for CTSK production, data related to main Figure 4. a** ECAR in cultured osteoclasts adherent on different substrates as indicated by the Seahorse assay in real-time. **b** The basal and maximal glycolysis levels in the ECAR shown in (a) were compared between osteoclasts adherent on collagen-coated substrates and plastic substrates (n = 6 for plastic, n = 3 for collagen-coating). One-way ANOVA. ****p* < 0.001, n.s., no significant difference. **c** CTSK protein levels and the basal glycolysis level in the ECAR in osteoclasts treated with doses of 3BrPA for 6 hours. One-way ANOVA. **p* < 0.05, ***p* < 0.01, ****p* < 0.001. **d** CTSK protein levels and the basal glycolysis level in the ECAR in osteoclasts treated with doses of 2DG for 6 hours. One-way ANOVA. **p* < 0.05, ***p* < 0.01, ****p* < 0.001. **e** Basal OCR and ECAR in osteoclasts treated with rotenone or oligomycin A. One-way ANOVA, ****p* < 0.001, n.s., no significant difference. **f** CTSK protein levels in osteoclasts treated with doses of rotenone or oligomycin A for 6 hours, respectively. **g** Immunofluorescence staining analysis to show the CTSK expression level in osteoclasts adherent on type I collagen-coated substrates. Osteoclasts were treated with 10 nM oligomycin A or 5 µM 2DG for 4 hours (scale bar, 50 µm, n = 5 for control, n = 3 for oligomycin A, n = 5 for 2DG). One-way ANOVA, *****p* < 0.0001, n.s., no significant difference. **h** Immunofluorescence staining analysis to show the CTSK expression level in osteoclasts adherent on type II collagen-coated substrates. Osteoclasts were treated with 10 nM oligomycin A or 5 µM 2DG for 4 hours (scale bar, 50 µm, n = 5 for control, n = 4 for oligomycin A, n = 5 for 2DG). One-way ANOVA, ****p* < 0.001, n.s., no significant difference. **i** Immunoblots of the pre-proenzyme and active CTSK levels in osteoclasts treated with 10 nM oligomycin A for 0, 2, 6 and 10 hours, respectively. **j** Immunoblots of pre-proenzyme and active CTSK levels in osteoclasts treated with 100 nM rotenone for 0, 2, 6 and 10 hours, respectively. All data are presented as mean ± SEM.

**Fig. S7 Osteoclast CTSK production is regulated by acetyl-CoA availability, data related to main Figure 5. a** Schematic of *ex vivo* experiment to study nutritional substrate fuels of CTSK expression. Osteoclasts were induced by RANKL and MCSF for 4 to 6 days followed by administration of different nutritional substrates or metabolites. **b** Immunoblots of the pre-proenzyme form and active CTSK form protein levels in osteoclasts grown in media with 10 mM pyruvate for increasing incubation times (0, 2 or 6 hours). **c** Immunoblots of the pre-proenzyme form and active CTSK form protein levels in osteoclasts grown in media with 10 mM glutamine for increasing incubation times (0, 2 or 6 hours). **d** Immunoblots of the pre-proenzyme and active CTSK forms, MMP-9 and ACP5 protein levels in osteoclasts grown in media with increasing DMKG concentrations for 6 hours, respectively. **e** Immunoblots of the pre-proenzyme form and active CTSK form protein levels in osteoclasts grown in media with increasing acetate concentrations for 6 hours, respectively. **f** Immunoblots of the pre-proenzyme and active CTSK forms, MMP-9 and ACP5 protein levels in osteoclasts grown in media with 1 mM acetate concentration for increasing incubation times (0, 2 or 6 hours). **g** Immunoblots of the pre-proenzyme form and active CTSK form protein levels in osteoclasts grown in media with increasing citrate concentrations for 6 hours, respectively. **h** Immunoblots of the pre-proenzyme and active CTSK forms, MMP-9 and ACP5 protein levels in osteoclasts grown in media with 100 µM citrate concentration for increasing incubation times (0, 2 or 6 hours). **i** RNA-seq analysis of the transcriptional level expression of ACSS1, ACSS2, ACSS3 and ACLY in Rheb1-deficient osteoclasts compared to controls (n = 4).

**Fig. S8 Excessive acetate supplementation suppresses CTSK production, data related to main Figure 5. a** Q-PCR analysis of the CTSK mRNA level in osteoclasts grown in media with 30 mM acetate for 6 hours. Unpaired t test, n.s., no significant difference. Data are presented as mean ± SEM. **b** Immunoblotting analysis of the CTSK protein expression level in osteoclasts grown in media with 30 mM acetate for 6 hours. **c** Immunoblots for H3K9ac, H3K14ac, H3K18ac, H3K27ac, H3K56ac and H3 with their quantitative densitometric results in osteoclasts. Cells were incubated in media with RANKL and M-CSF for 4 days and administrated with or without acetate for 6 hours before collection. Membrane-impermeant acetyl-CoA trisodium was a negative control.

**Fig. S9 Additional acetate restored the SirT3 level in Rheb1-deficient osteoclast-like cells, data related to main Figure 5. a** Mitochondrial SirT3 protein level was determined by immunoblotting in Rheb1-deficient osteoclasts compared to controls, with or without 1 mM acetate addition for 6 hours, respectively. Tom20 was used as an internal control. **b** Immunoblotting of Rheb1 and pS6 expression levels in the Raw264.7 cell line with stable knockout of the Rheb1 gene, with α-tubulin as an internal control. **c** Mitochondrial SirT3 protein level was determined by immunoblotting in RANKL-incubated Rheb1 KO Raw264.7 and wildtype controls, with or without 10 mM acetate addition for 3 hours, tom20 was used as an internal control. **d** Mitochondrial SirT3 protein level was determined by immunoblotting in Rheb1 KO Raw264.7 and wildtype controls, with or without 10 mM acetate addition for 3 hours, with tom20 as an internal control.

**Fig. S10 Alcohol consumption delays bone fracture healing, data related to main Figure 6. a** Body weight in mice with alcohol consumption during development in comparison to mice feeding normally (n = 10 for control diet, n = 10 for 1% and n = 12 for 5% alcohol consumption) **b** Body weight changes in mice with or without alcohol consumption in (a). One-way ANOVA, n.s., no significant difference. **c** Serum CTX-I and P1NP levels in alcohol consumption model in litters of mice (n = 7 for control diet, n = 8 for others). One-way ANOVA, n.s., no significant difference. **d** Images of drill-hole in mice with 1% or 3% alcohol consumption in comparison to mice feeding normally. **e** Body weight changes in adult mice with alcohol consumption in comparison to mice feeding normally (n = 7). One-way ANOVA, n.s., no significant difference. **f** Serum TNF-ɑ and IL-6 levels in adult mice with alcohol consumption in comparison to mice feeding normally (n = 8 for control diet, n = 7 for 1% and n = 8 for 3% alcohol consumption). One-way ANOVA, n.s., no significant difference. **g** Immunoblot analysis of the CTSK protein level in heart tissues from mice with 1% or 3% alcohol consumption. **h** Serum ethanol concentration in drill-hole surgery Rheb1^OC^ mice compared to that in littermate controls in the context of alcohol consumption. One-way ANOVA, **p* < 0.05, ***p* < 0.001. **i** Serum TNF-ɑ and IL-6 levels in drill-hole surgery Rheb1^OC^ mice compared to those in littermate controls in the context of alcohol consumption. One-way ANOVA, n.s., no significant difference. All data are presented as mean ± SEM.

**Fig. S11 Integrin signaling is not involved in regulating CTSK. a** Immunoblots of CTSK levels in osteoclasts adherent to type I collagen-coated substrates compared to cells cultured on plastic substrates, with or without treatment 10 µM integrin α_v_β_3_ inhibitor (Cyclo(-RGDfK)) for 6 hours. ɑ-tubulin was used as an internal control. **b** Immunoblots of integrin levels in osteoclasts treated with OxPhos inhibitors for 6 hours, respectively. ɑ-tubulin was used as an internal control. **c** Immunoblots of SirT3 levels in osteoclasts treated with Cyclo(-RGDfK) for 6 hours, with TOM20 as an internal control.

Table 1. Primers used in PCR and Q-PCR

| Oligonucleotides | Source | Identifier | |
| --- | --- | --- | --- |
| *Rheb1*-Flox(F)-genotyping primer  GCCCAGAACATCTGTTCCAT | doi.org/10.1016/j.devcel.2010.11.020 | | N/A |
| *Rheb1*-Flox(R)-genotyping primer  GGTACCCACAACCTGACACC | doi.org/10.1016/j.devcel.2010.11.020 | | N/A |
| *CTSK*-Cre(F)-genotyping primer  TTATTCCTTCCGCCAGGATG | doi.org/10.1016/j.cell.2007.07.025 | | N/A |
| *CTSK*-Cre(R)-genotyping primer  TAGTTTTTACTGCCAGACCG | doi.org/10.1016/j.cell.2007.07.025 | | N/A |
| *Raptor*-Flox(F)-genotyping primer  CTCAGTAGTGGTATGTGCTCAG | The Jackson Laboratory | | N/A |
| *Raptor*-Flox(F)-genotyping primer  GGGTACAGTATGTCAGCACAG | The Jackson Laboratory | | N/A |
| *CTSK*-RT-F  TGAACCATGCAGTGTTGGTG | This paper | | N/A |
| *CTSK*-RT-R  TATTCCGAGCCAAGAGAGCA | This paper | | N/A |
| *MMP-9*-RT-F  CAAAGACCTGAAAACCTCCAAC | This paper | | N/A |
| *MMP-9*-RT-R  GACTGCTTCTCTCCCATCATC | This paper | | N/A |
| *ACP5*-RT-F  AGCAGCCAAGGAGGACTACG | This paper | | N/A |
| *ACP5*-RT-R  TGGCTAACAATGGTCGCAAG | This paper | | N/A |
| *Nfatc1*-RT-F  GGCTGGTCTTCCGAGTTCACATC | This paper | | N/A |
| *Nfatc1*-RT-R  GCTGTCTGTGCTCTGCTTCTCC | This paper | | N/A |
| *Rheb1*-RT-F  CGGTCTGTGGGAAAGTCCTC | This paper | | N/A |
| *Rheb1*-RT-R  ATATTCATCCTGCCCCGCTG | This paper | | N/A |
| *B2M*-RT-F  CTGCTACGTAACACAGTTCCACCC | This paper | | N/A |
| *B2M-*RT-R  CATGATGCTTGATCACATGTCTCG | This paper | | N/A |

**Supplemental Materials and Methods**

**Cell Mito stress test and glycolysis stress test seahorse assays**

The Agilent Seahorse XF Cell Mito Stress Test kit (Seahorse XFe96/XF Pro FluxPak, 103793-100, Agilent Technologies) was used for the assays. The OCR and ECAR were determined on a Seahorse XFe96 Analyzer (Agilent Technologies). Briefly, osteoclasts were seeded in the Agilent Seahorse XFe96 Spheroid Microplate wells. The day before the assay, a sensor cartridge was hydrated in double-distilled water at 37 °C in a non-CO_2_ incubator overnight. On the day of the assay, the Seahorse XF Calibrant was replaced at 37 °C in a non-CO_2_ incubator for a minimum of 1 hour. The substrate-limited medium was replaced with assay medium composed of unbuffered XF DMEM (103575-100, Agilent Technologies) supplemented with 10 mM glucose (103577-100, Agilent Technologies), 1 mM pyruvate (103578-100, Agilent Technologies), 2 mM glutamine (103579-100, Agilent Technologies), 50 ng/mL RANKL and 20 ng/mL M-CSF. The cells were equilibrated for 1 h at 37 °C in a CO_2_-free incubator. For the Mito Stress Test, the OCR measurement cycles consisted of 3 minutes of mixing and 3 minutes of measurement. Following three baseline OCR measurement cycles, oligomycin (1 µM, Agilent Technologies), FCCP (2 µM, Agilent Technologies), and a mixture of rotenone (1 μM, Agilent Technologies) and antimycin A (1 µM, Agilent Technologies) were sequentially injected every three cycles. The mitochondrial stress test experiment measured the OCR by sequentially adding targeted drugs to the mitochondrial electron transport chain to obtain key parameters reflecting mitochondrial function. For the Glycolysis Stress Test, only glutamine (2 mM) was added to the base medium, and the steps were the same as the OCR measurement cycles, except for the additions. Following three baseline ECAR measurement cycles, glucose (150 mM), oligomycin (1 µM), and 2-DG (50 mM, Agilent Technologies) were added in order every three cycles. Measurement of the OCR and ECAR were calculated during the Mitostress test by the Seahorse XFe96 software, Wave 2.6.1.

**Metabolite measurement**

Osteoclasts were seeded at 5×10^4^ cells/well in 24-well plates. On the day of the assay, cells were disrupted using ultrasonic waves (200 W, in an ice bath) for 3 seconds with a 10-second interval, centrifuged at 8000×*g* for 10 minutes at room temperature and the supernatant collected for testing. The cellular concentrations of pyruvate (BC2200, Solarbio), acetyl-CoA (E-EL0125c, Elabscience, Wuhan, China) or lactate (BC2230, Solarbio) were determined using Colorimetric Assay Kits according to the manufacturers’ instructions and adjusted for cell counts.

**Drill-hole injury model**

Ten-week-old male mice were utilized to establish a femoral drill hole model^1^. Anesthesia was induced using 1.25% Avertin, and the mice were placed on a sterile surgical field. The periosteum was scratched lengthwise in the middle of the anteromedial femur using a needle tip. A circular bone defect, 0.8 mm in diameter, was then created at the mid-diaphysis of the femur using a micro-drill. Care was taken to ensure that the drilling direction was perpendicular to the coronal plane of the femur to prevent damage to adjacent structures. After creating the defect, saline was applied to cool the drill site and flush out bone debris. The muscle and skin layers were subsequently sutured with absorbable sutures.

**Lentivirus transfection**

Lentivirus transduction of osteoclast was performed on the middle-stage of osteoclast differentiation, with addition of 5μg/ml polybrene. The coding sequence of Rheb-NM_053075 was synthesized and cloned into the GYVB218 vector using restriction sites BamHI/Agel. The vector elements were arranged as Ubi-MCS-EGFP-IRES-Puromycin. Overexpressed plasmid was produced by Geneyuan (Guangzhou, People’s Republic of China).

**Micro-computed tomography (micro-CT) analysis**

Femurs from 10–12-week-old male mice (Rheb1^OC^ mice and littermate controls) were scanned using a Scanco μCT80 scanner (Scanco Medical AG, Bassersdorf, Switzerland) at a resolution of 10 μm, and calibrated three-dimensional images were reconstructed. In total, 200 slices were analyzed for all scans. For distal femur bone microarchitecture analysis, trabecular bone was prepared to exclude the cortical bone, then cancellous bone parameters, including the bone mineral content, bone volume/tissue volume, trabecular number, trabecular separation/spacing and trabecular thickness, were determined. For the drill-hole femur, 200 slices were analyzed, starting with the first slice in which bone callus were no longer visible, likewise, ending with the final slice in which bone callus were no longer visible.

**Measurement of bone resorptive activity ex vivo**

Osteoclasts were seeded on bone slices (AE-80100, Immunodiognosticsystems, Rochester, USA) with M-CSF and RANKL incubation. At 2 days prior to determination, the culture medium was replaced, and cell supernatants were collected to determine CTX-I (MBS263207, MyBioSource, San Diego, USA) and TRAP5b (ELK9616, ELK Biotechnology, Wuhan, China) concentrations. For the MMP-9 activity assay, cell homogenates and cell supernatants were mixed to determine the protein level (E-ELM3052, Elabscience, Wuhan, China), the enzymatic activity was subsequently tested by gelatin enzymography (RTD6143, Real-Times, Beijing, China) using the protein concentration as the internal control. All measurements were performed according to the manufacturers’ instructions.

**Measurement of bone turnover markers in vivo**

The serum CTX-I (MBS263207, MyBioSource, San Diego, USA) and P1NP (ELK5446, ELK Biotechnology, Wuhan, China) concentrations were determined by ELISA according to the manufacturers’ instructions.

**Bone histomorphometry**

Whole-mount skeletal preparations were stained using Alizarin Red and Alcian Blue stain following previously described steps^2^. For bone section staining, bone samples were resected and fixed in 10% neutral-buffered formalin overnight, followed by decalcification in 10% EDTA (pH 7.3) and paraffin embedding. Sections of 10 μm thickness were processed for staining for TRAP following a standard protocol (387A-1KT, Sigma-Aldrich), and TRAP-positive osteoclasts were counted using Image J. Sections of 3 μm thickness were processed for hematoxylin and eosin (H&E) or immunofluorescence staining. For immunofluorescence staining, anti-Col1a1 primary antibody was used at a dilution of 1:100 (501352, ZEN BIO, Beijing, China) and anti-Col2 primary antibody at a dilution of 1:100 (ab34712, Abcam, Cambridge, UK). Un-decalcified frozen bone sections of 20 μm thickness were prepared to show the growth plate mineralized matrix. Section staining was performed according to the manufacturer’s instructions of the von Kossa stain kit (ab150687, Abcam). H&E- or von Kossa-stained sections or sections stained for TRAP were scanned by transmitted-light microscopy (Axio Scope A1, ZEISS, Japan) and representative images were acquired using ZEN 2011 (ZEISS, Japan). Sections stained with immunofluorescence were scanned using a FV1000 confocal laser microscope (Olympus, Tokyo, Japan). For all evaluations, the stained slides were scored using ImageJ.

**Immunoblotting**

Tissues and cell lysates were prepared in 2×SDS lysis buffer supplemented with protease inhibitor cocktail (05892791001, Roche, Cornwall, UK). For CTSK and SirT3 detection, lysates were separated by SDS-PAGE immediately followed by western blotting. Antigen detection was performed using primary antibodies to CTSK (1:1500, sc-48353, Santa Cruz, California, USA), MMP9 (1:2000, ab38898, Abcam, UK), ACP5 (1:1000, M03277-1, Boster Biological Technology, Wuhan, China), type I collagen (1:1000, PTM-6219, PTM Bio, Hangzhou, China), type II collagen (1:1000, ab188570, Abcam), Rheb (1:1000, sc-271509, Santa Cruz, USA), phospho-S6 (1:3000, #2211, Cell Signaling Technology, Boston, USA), S6 (1:3000, sc-74459, Santa Cruz), CTSB (1:800, YT0679, ImmunoWay, Texas, USA), SQSTM1/p62 (1:1500, #23214, Cell Signaling Technology), acetyl-histone H3 (1:1000, #9927, Cell Signaling Technology), SirT3 (1:1000, #5490, Cell Signaling Technology), Integrin β3 (1:500, A19073, ABclonal, MA, USA), TOM20 (1:1000, #42406, Cell Signaling Technology) or α-tubulin (1:5000, RM2007, Ray Antibody Biotech, Beijing, China).

**Rheb1 stable knockout cell line establishment**

The Rheb1 knock-out Raw264.7 cell line was generated based on the CRISPR-Cas9 system. Four pairs of small guide RNA were designed to target exon 3:

gRNA1: AAGTTGTGACAAGACTATTAGGG,

gRNA2: GACAGAACATTAACCTTCTGTGG,

gRNA3: TCTCATCAGCAATTCCTAGCAGG,

gRNA4: TCACACACACTCGGGGTGTCAGG.

Electroporation was performed for transfection. We detected the expression of Rheb1 protein level changes of the genetically modified cells.

**ROS assay**

ROS detection was performed according to the manufacturer’s instructions (HB220909, Yeasen, Shanghai, China). Briefly, cells were seeded in the presence of M-CSF and RANKL. After washing the cells twice with sterile PBS, 10 μM Dichloro-dihydro-fluorescein diacetate (DCFH-DA) was added and counterstained at 37 °C in the dark for 30 minutes. Subsequently, the cells were washed three times with serum-free medium to minimize interference with excess DCFH-DA. Dichloro-dihydro-fluorescein (DCF) fluorescence intensity was determined by flow cytometry (CytExpert, Beckman, California, USA).

**Mito-tracker and lyso-tracker assays**

BMDMs were cultured in a glass bottom confocal dish, cells were osteoclast-induced with 25 ng/mL M-CSF and 100 ng/mL RANKL and multinucleated cells were formed. On the day of the assay, cells were incubated with Mito-Tracker Red CMXRos (C1049B, Yeasen) and LysoTracker Green DND-26 (40738ES50, Yeasen) diluted in fresh replaced cultured medium in the presence of M-CSF and RANKL. After 30 minutes of incubation, the cells were washed twice with PBS and the culture medium was replaced with ɑ-MEM supplemented with M-CSF and RANKL. Images were obtained with an FV1000 confocal laser microscope (Olympus). Mitophagy was quantified using Image J by colocalization between dyes for the mitochondrion and lysosome in microscopy images.

For assays on bone slices, BMDMs were plated on cortical bovine bone slices of 200 µm (DT-1BON1000-96-1, Immunodiagnostic Systems, Maryland, USA) in 96-well plates with M-CSF and RANKL induction. When multinucleated cells had formed, they were incubated with Mito-tracker to determine mitochondria abundance. Images were obtained with an FV1000 confocal laser microscope (Olympus).

**Mitochondria isolation**

Mitochondria from osteoclasts or RANKL-incubated Raw264.7 were isolated according to the manufacturer’s instructions of a mitochondrial isolation kit (SM0020, Solarbio). The isolated pellet was resuspended and lysed in 2×SDS lysis buffer supplemented with protease inhibitor cocktail (05892791001, Roche) for immunoblotting determination. For SirT3 level detection, TOM20 was used as an internal control.

**Detection of Serum Biochemical Parameters**

Serum concentrations of IL-6 (ELK1157, ELK Biotechnology, Wuhan, China), TNFα (ELK1387, ELK Biotechnology), and ethanol (BC6035, Solarbio) were measured following the manufacturer’s protocols.

**RNA-Seq library preparation, sequencing, and analysis**

For transcriptional profiling of osteoclasts, BMDMs were induced with M-CSF and RANKL until most cells formed osteoclasts. An RNA-Seq library was prepared using the NEBNext^®^ Ultra™ RNA Library Prep Kit for Illumina^®^ (NEB) and sequenced with the Illumina NovaSeq 6000 (Illumina, San Diego, California, USA) sequencing platform (performed at the Novogene Corporation, Beijing, China). The subsequent analysis, including quality trimming, was executed using the BioSAILs workflow execution system. Fastp (v0.23.4) was used for quality trimming of the raw reads to omit low-quality bases, systematic base-calling errors, and sequencing adapter contamination. The quality of the sequenced reads pre/post quality trimming was assessed using FastQC (v0.12.0) and only the reads that passed quality trimming in pairs were retained for downstream analysis. The quality-trimmed RNA-Seq reads were aligned to the mouse transcriptome (*Mus musculus* GRCm39.109) using HISAT2 (v2.2.1). The conversion and sorting of SAM alignment files for each sequenced sample to the BAM format were performed using SAMtools (v0.1.18). The BAM alignment files were processed using FeatureCounts (v2.0.6), using the reference annotation file to produce raw counts for each sample. The raw counts were analyzed by DESeq2 (v1.40.2) to quantify gene expression with principal-component analysis, hierarchical clustering, and differential expression analysis, and differentially expressed genes (DEGs) were selected. DEGs by at least twofold (log2(FC) ≥ 2 and adjusted p-value of < 0.05 for upregulated genes, and log2(FC) ≤ −2 and adjusted p-value of < 0.05 for downregulated genes) between the osteoclasts derived from control and conditional knockout mice were subjected to Gene Ontology and Kyoto Encyclopedia of Genes and Genomes enrichment using the clusterProfiler (v4.8.3).

**References**

1. Inoue, S., Takito, J. & Nakamura, M. Site-Specific Fracture Healing: Comparison between Diaphysis and Metaphysis in the Mouse Long Bone. *Int. J. Mol. Sci.* **22**, (2021).

2. Mead, T. J. Alizarin Red and Alcian Blue Preparations to Visualize the Skeleton. *Methods Mol Biol*. **2043**, 207-212 (2020).
