# Supplementary figures and images for: Osteoclasts control endochondral ossification via regulating acetyl-CoA availability
Source: Bone Res. 2024 Aug 28;12:49. doi: 10.1038/s41413-024-00360-6 (PMC11358419; doi:10.1038/s41413-024-00360-6)

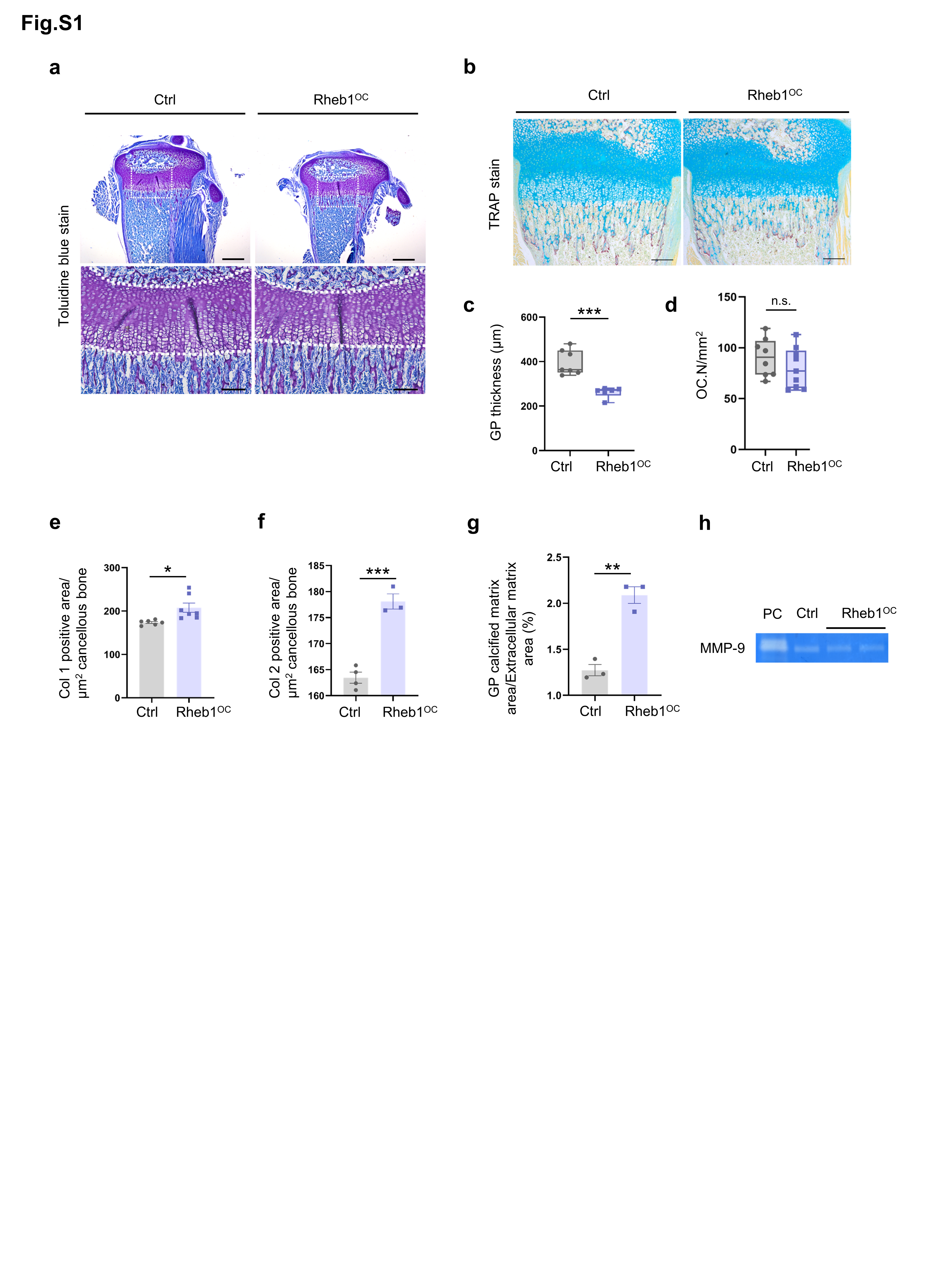

Supplement: Supplementary file 2 — Supplemental Figure 1 [file 41413_2024_360_MOESM2_ESM.tif]

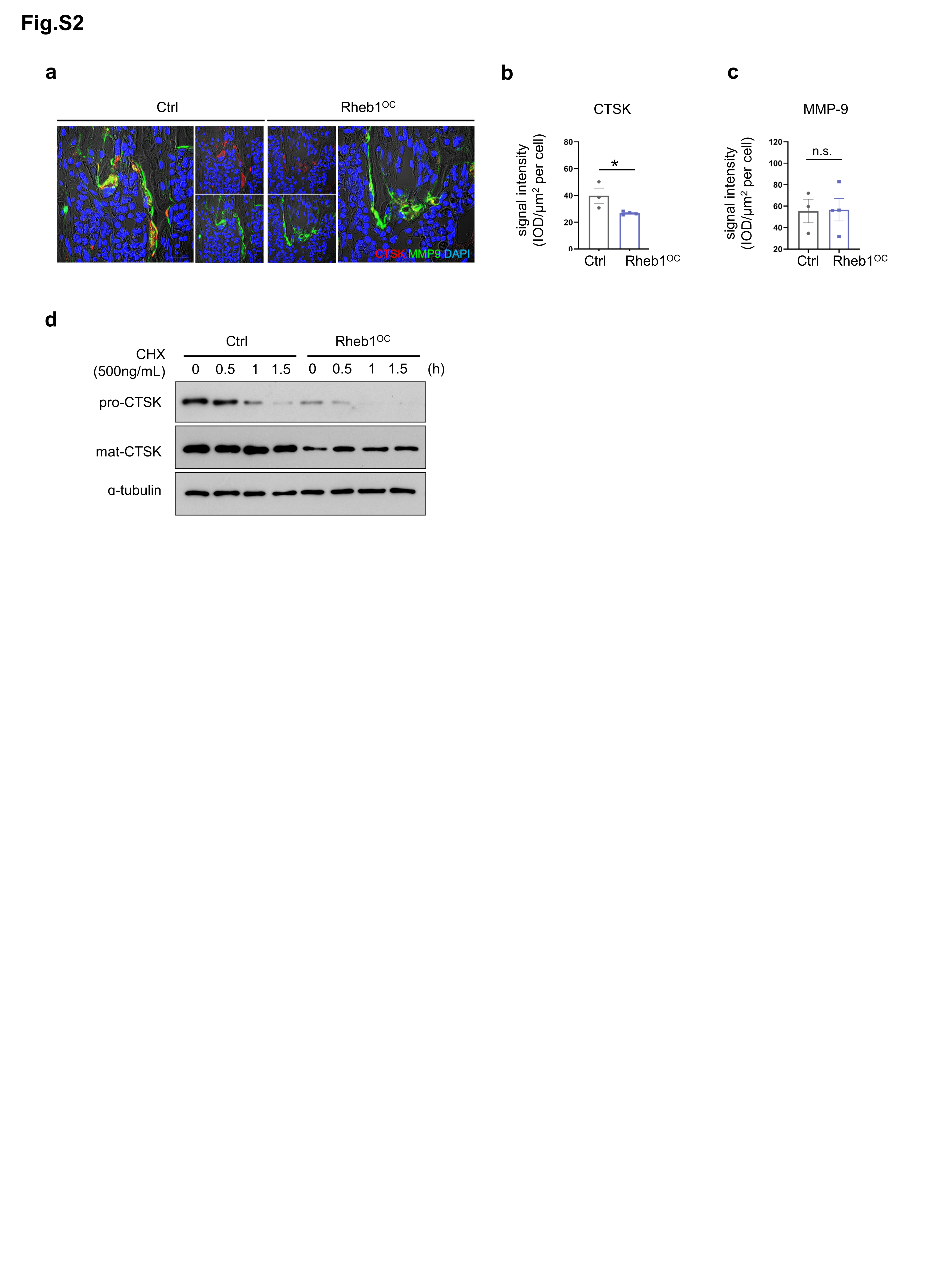

Supplement: Supplementary file 3 — Supplemental Figure 2 [file 41413_2024_360_MOESM3_ESM.tif]

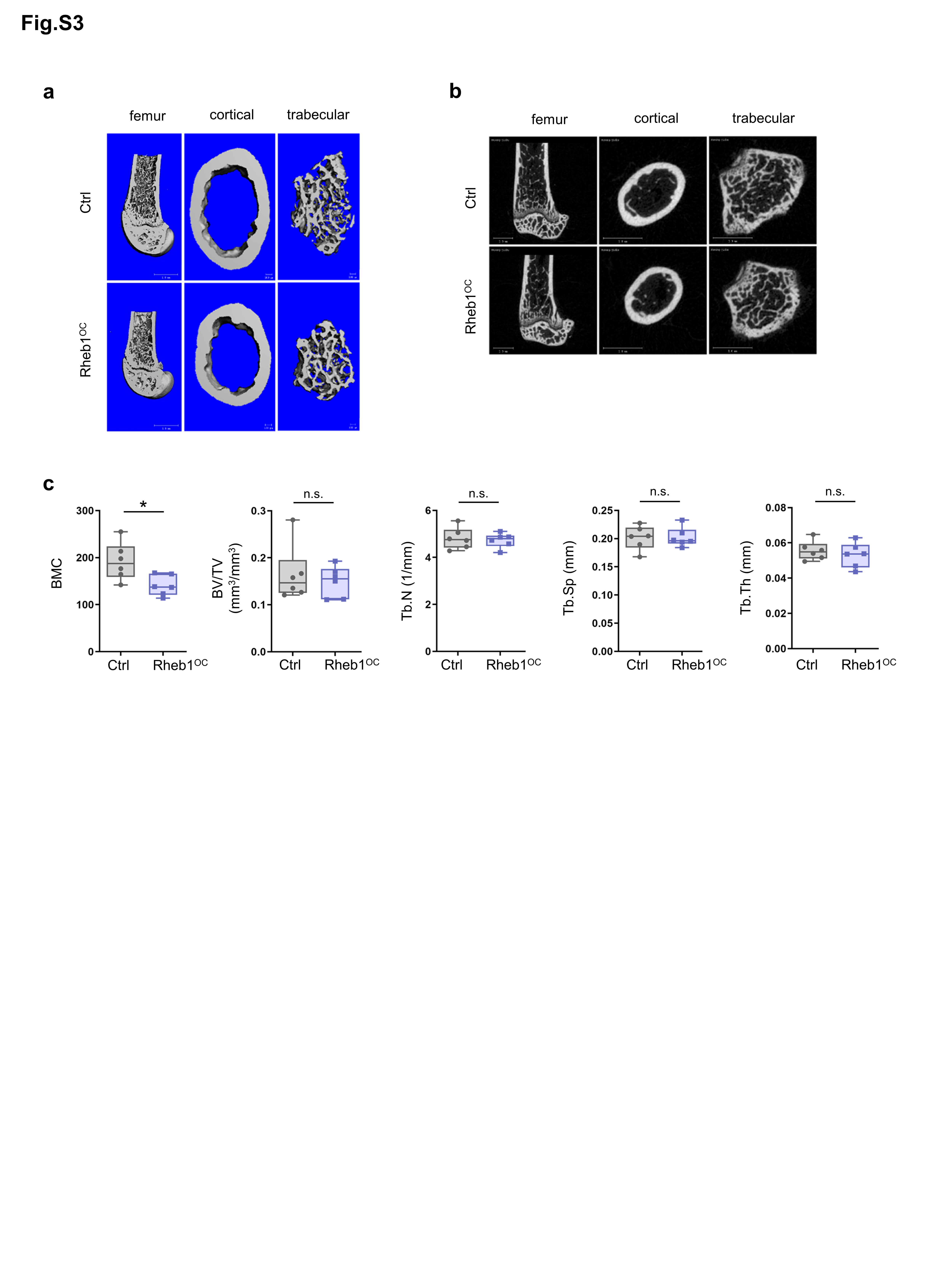

Supplement: Supplementary file 4 — Supplemental Figure 3 [file 41413_2024_360_MOESM4_ESM.tif]

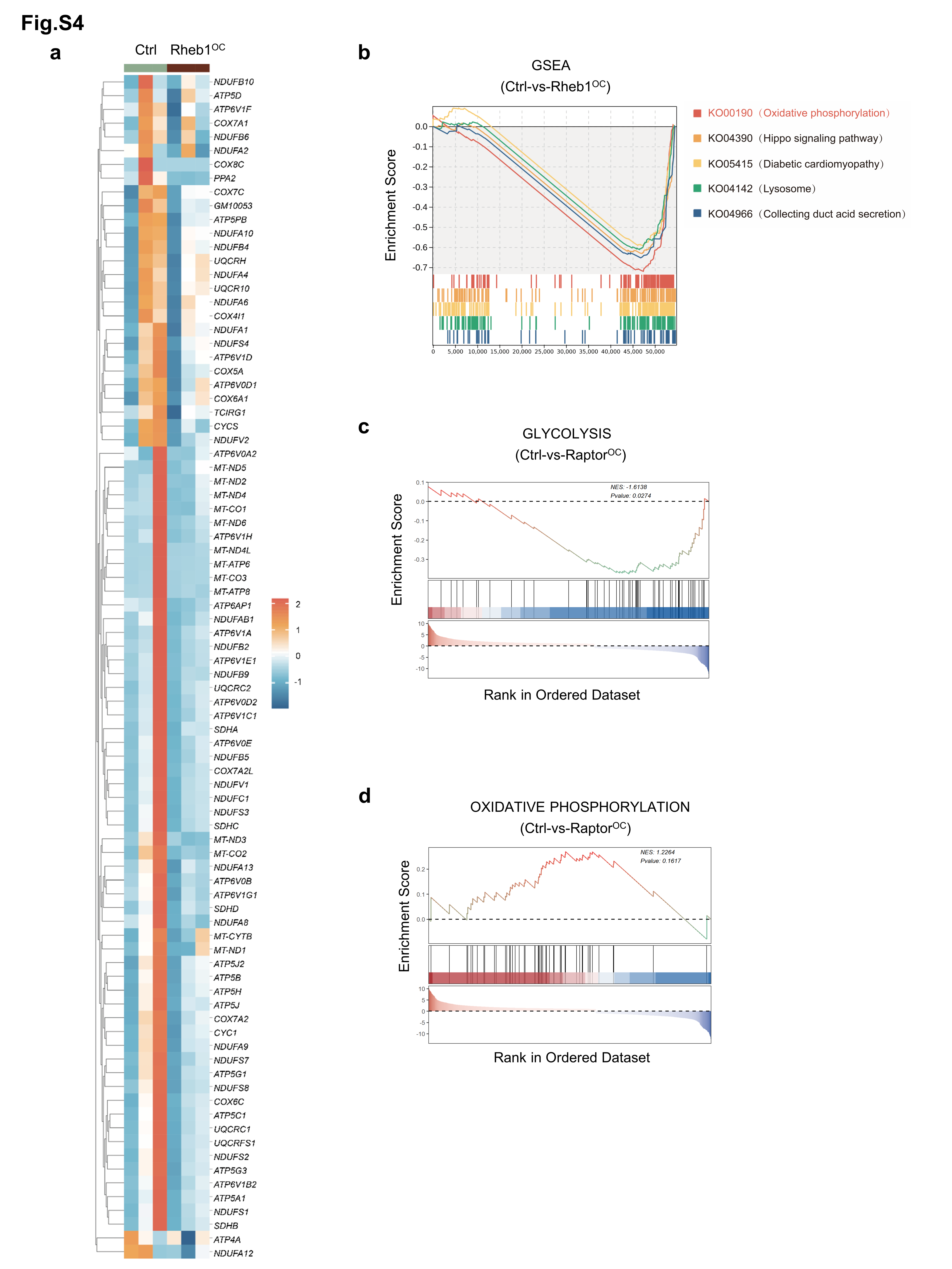

Supplement: Supplementary file 5 — Supplemental Figure 4 [file 41413_2024_360_MOESM5_ESM.tif]

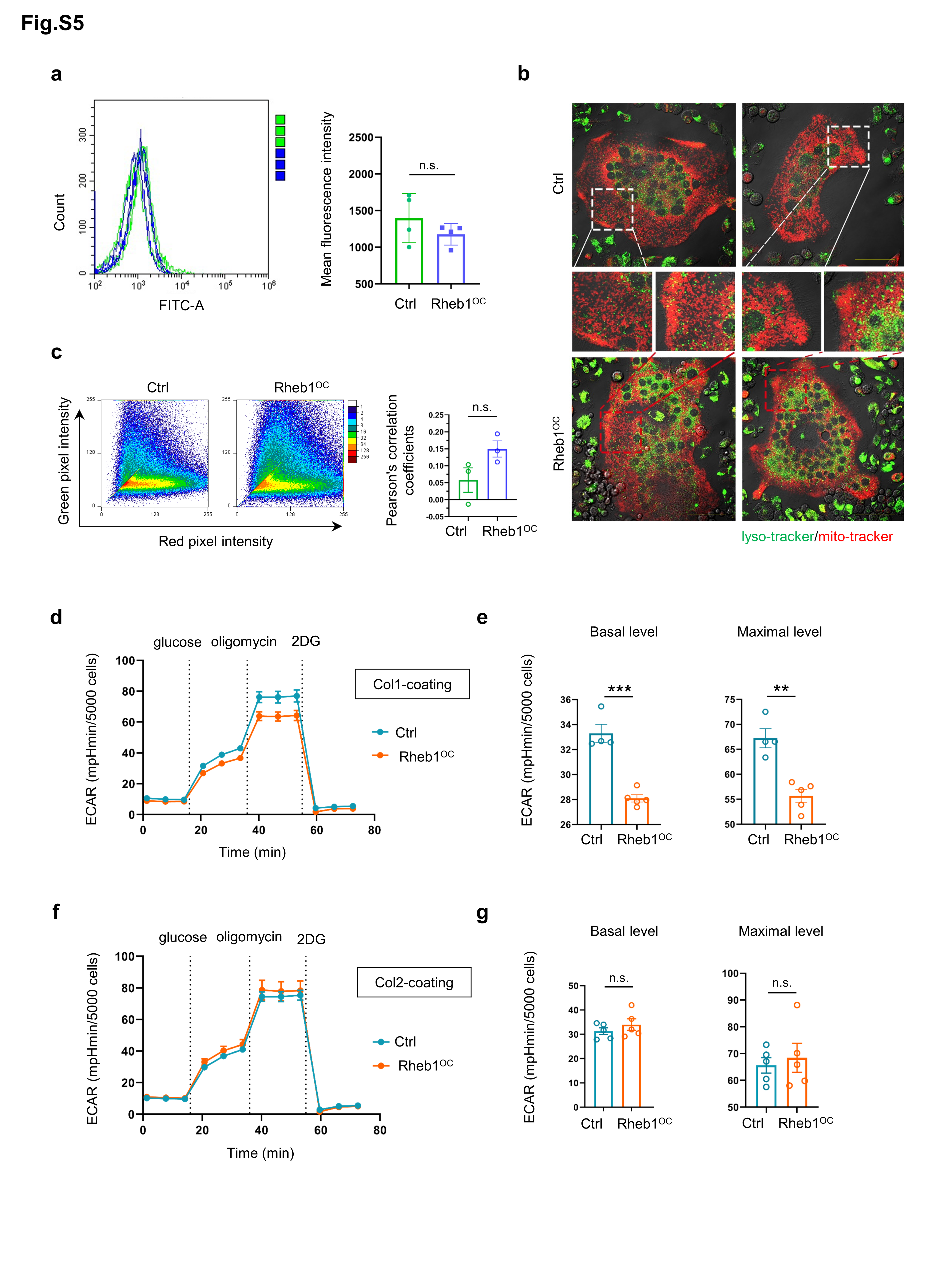

Supplement: Supplementary file 6 — Supplemental Figure 5 [file 41413_2024_360_MOESM6_ESM.tif]

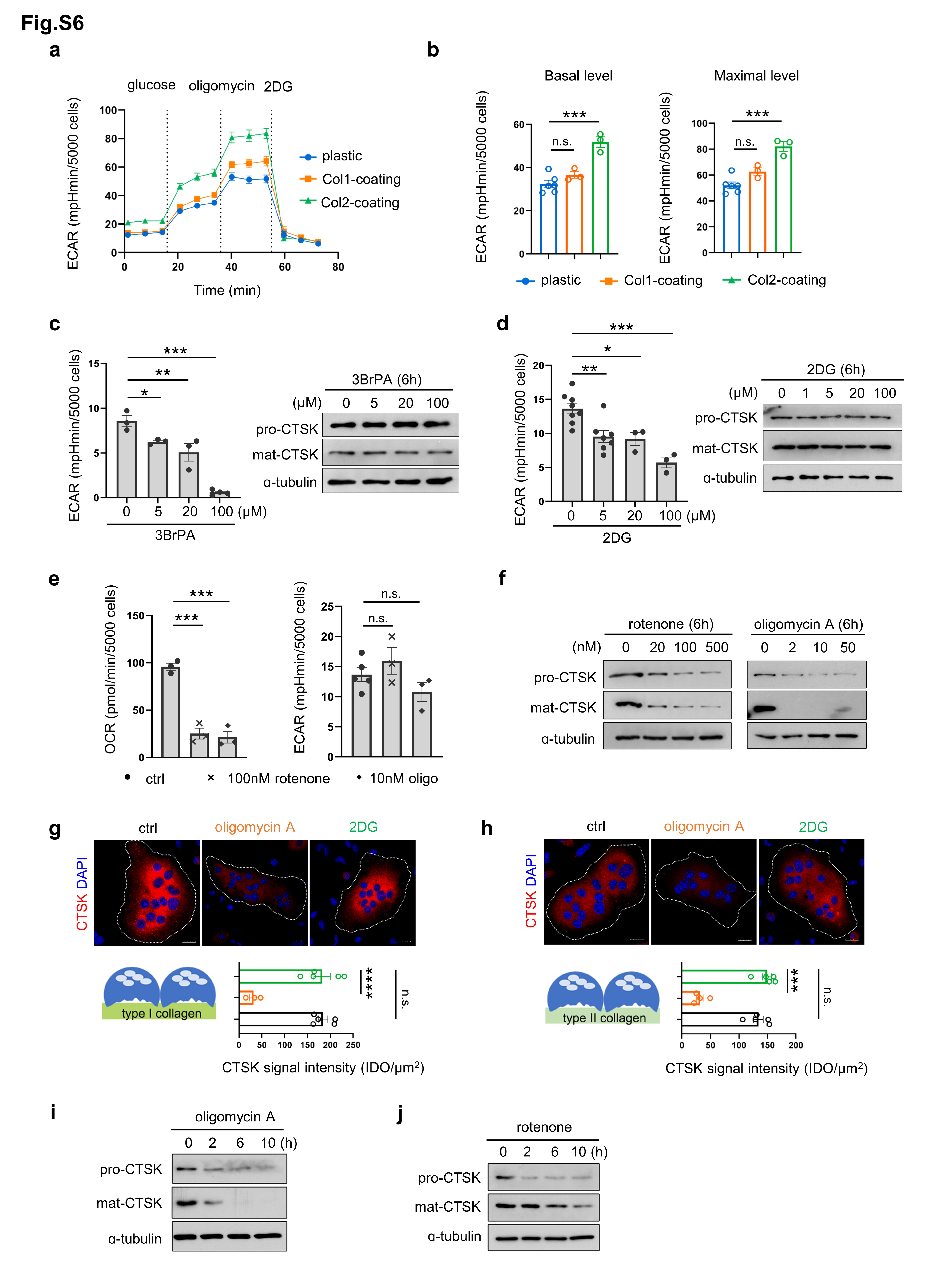

Supplement: Supplementary file 7 — Supplemental Figure 6 [file 41413_2024_360_MOESM7_ESM.tif]

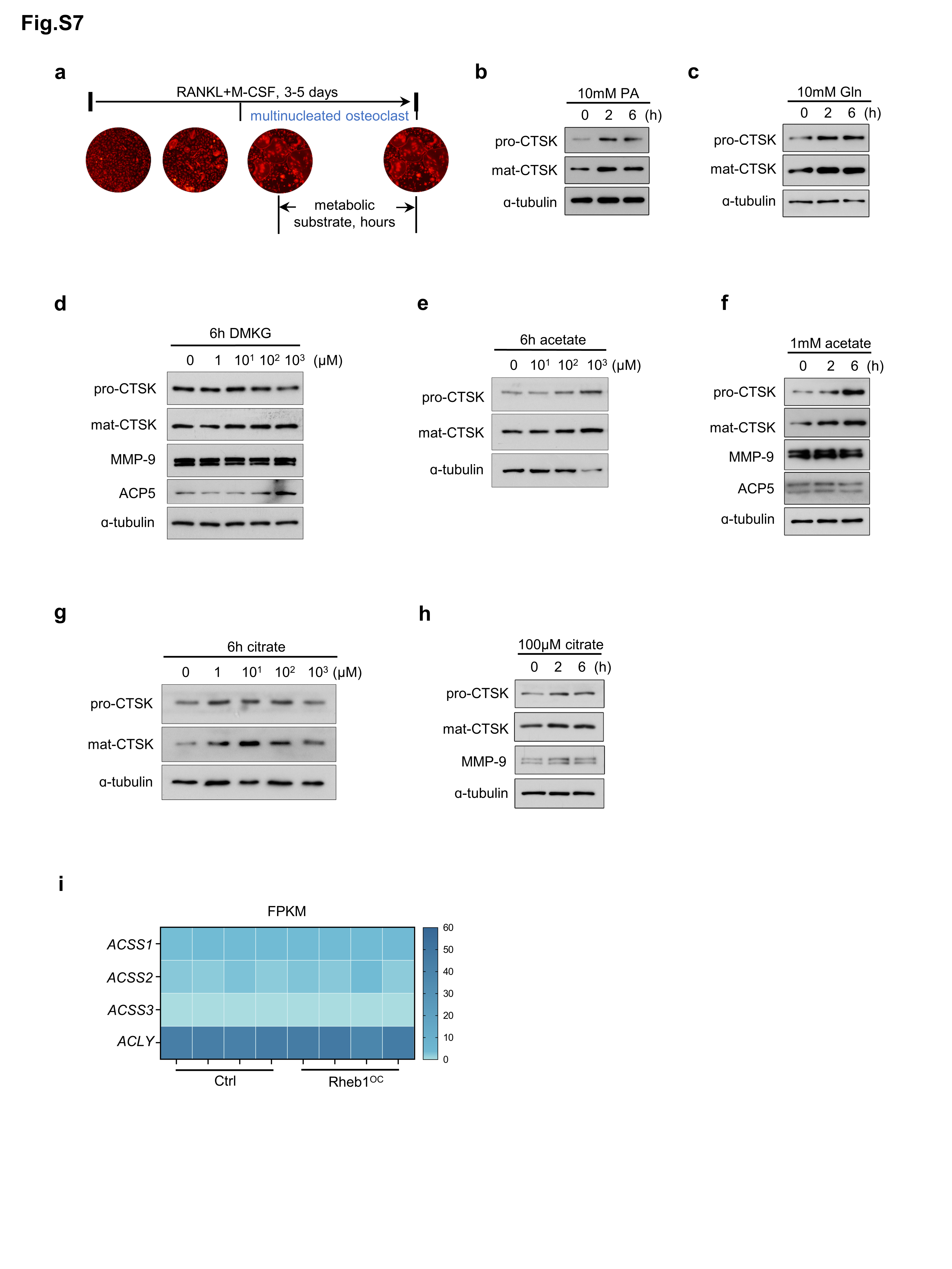

Supplement: Supplementary file 8 — Supplemental Figure 7 [file 41413_2024_360_MOESM8_ESM.tif]

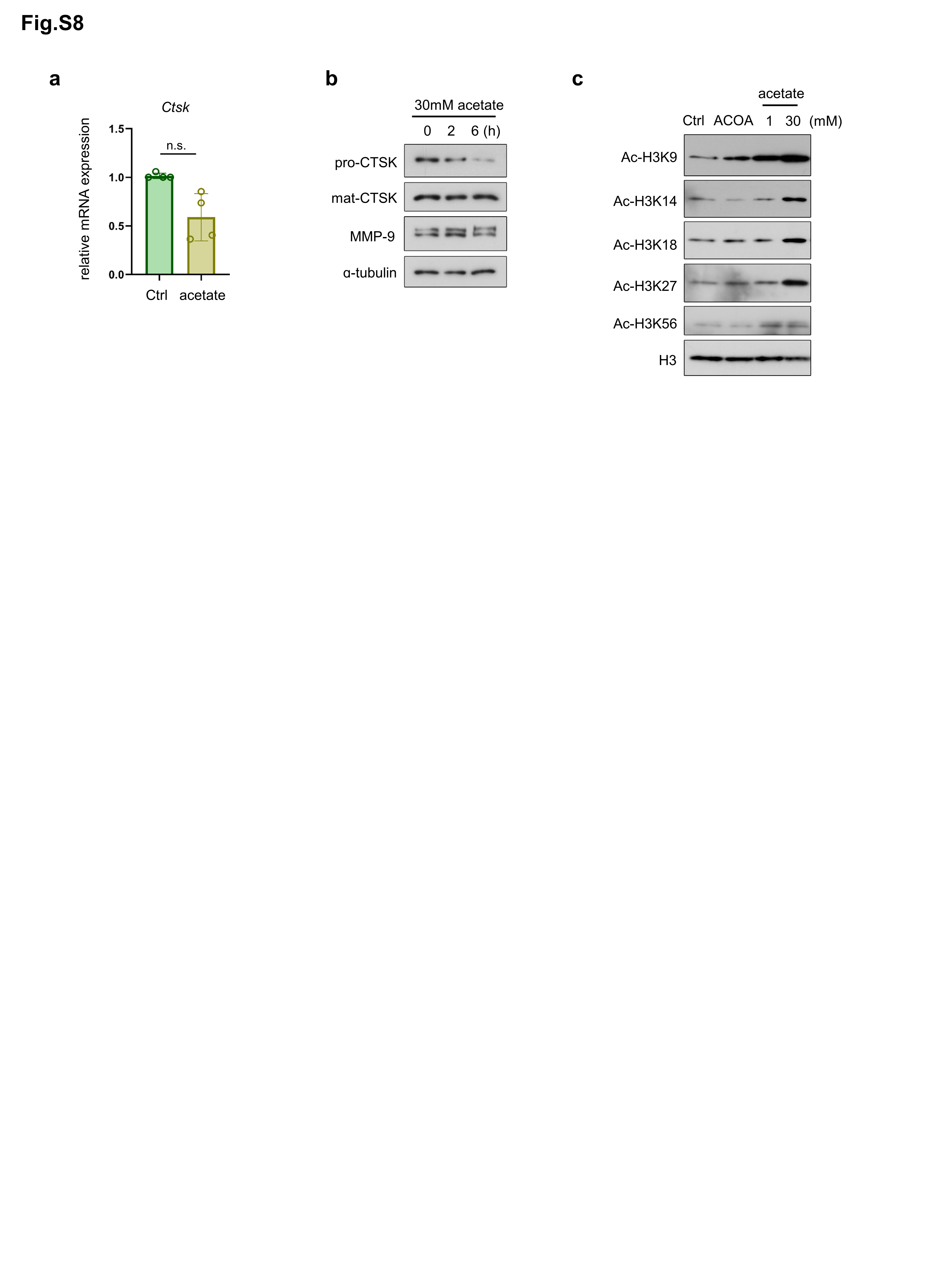

Supplement: Supplementary file 9 — Supplemental Figure 8 [file 41413_2024_360_MOESM9_ESM.tif]

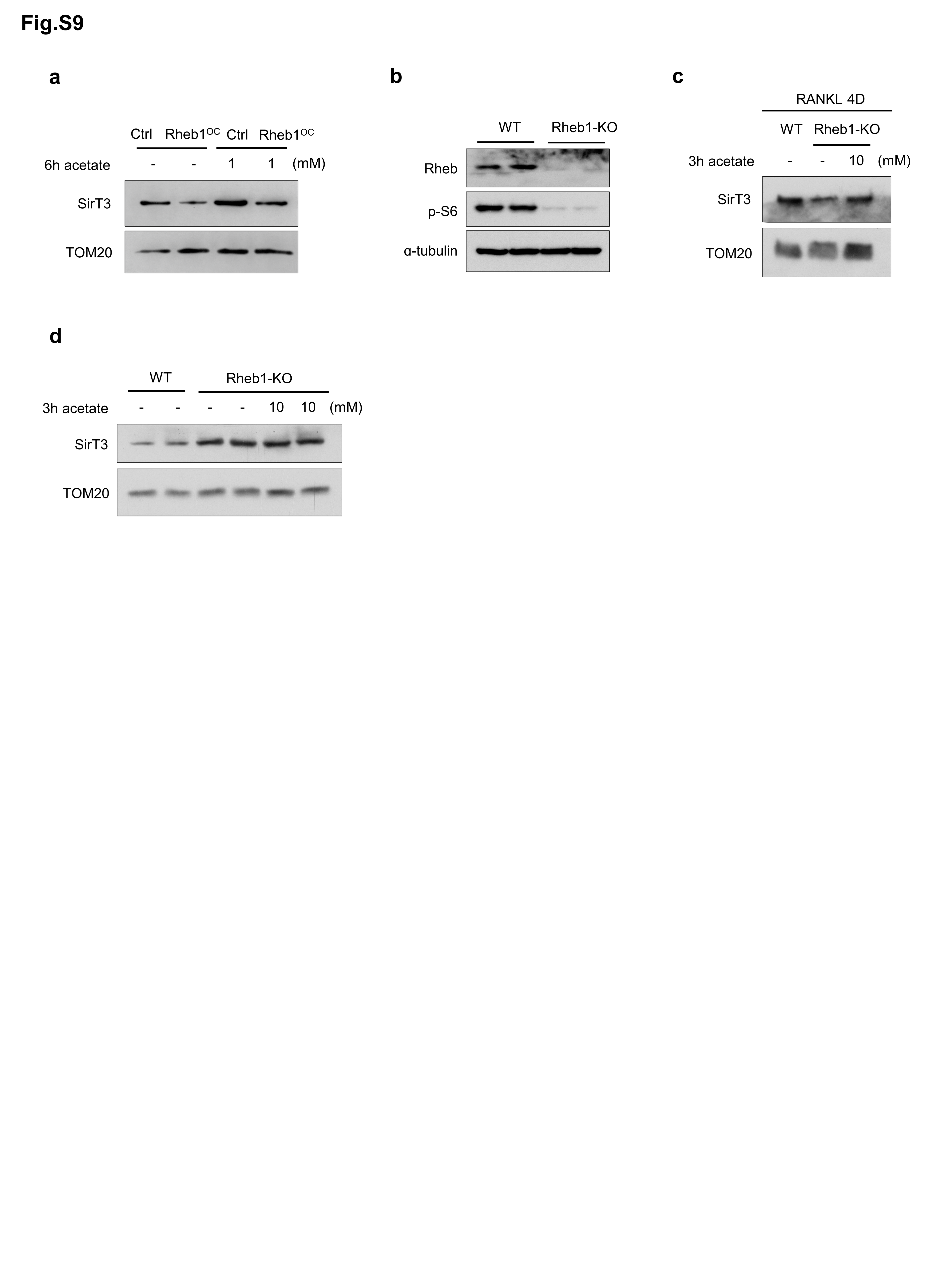

Supplement: Supplementary file 10 — Supplemental Figure 9 [file 41413_2024_360_MOESM10_ESM.tif]

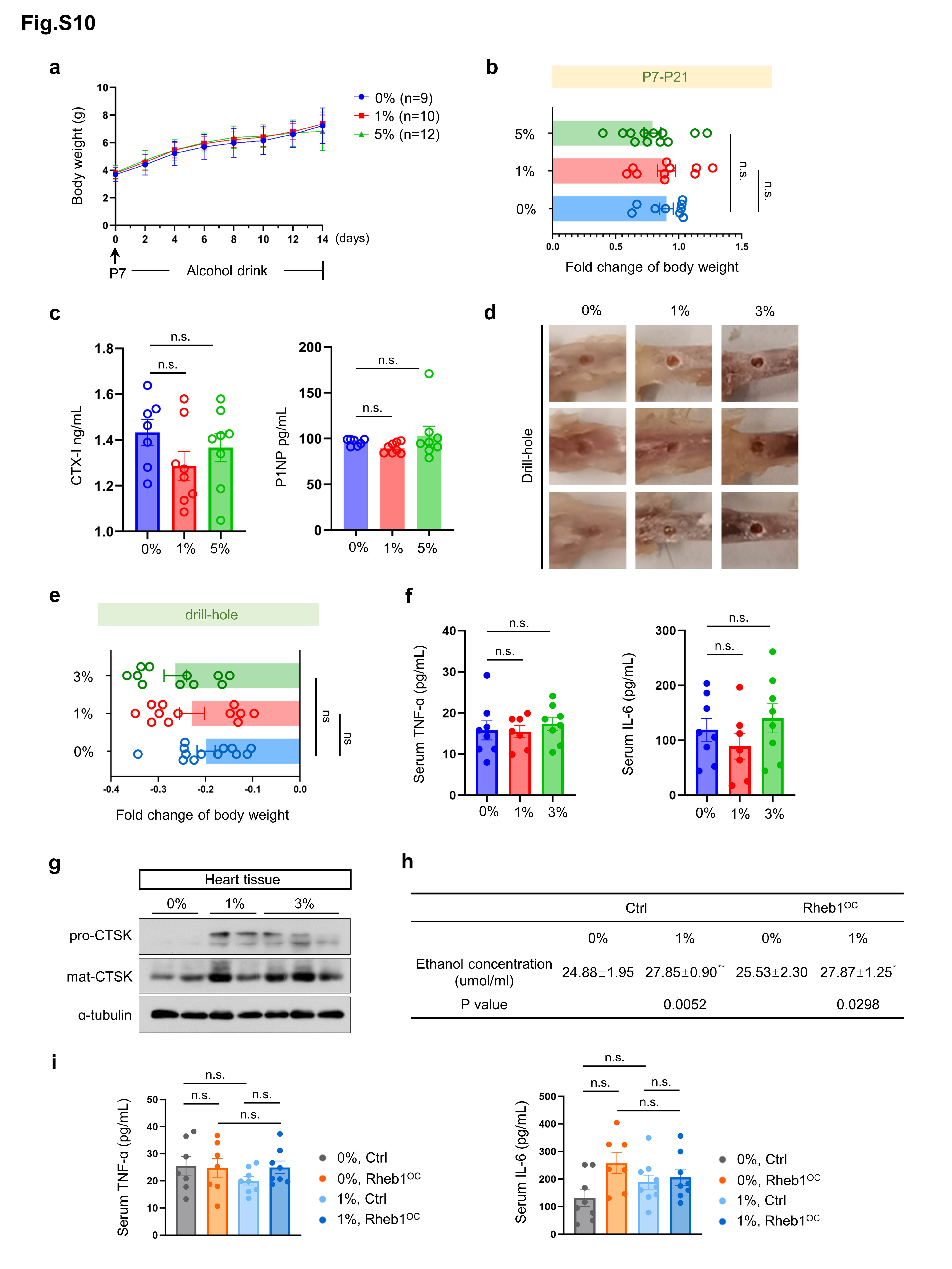

Supplement: Supplementary file 11 — Supplemental Figure 10 [file 41413_2024_360_MOESM11_ESM.tif]

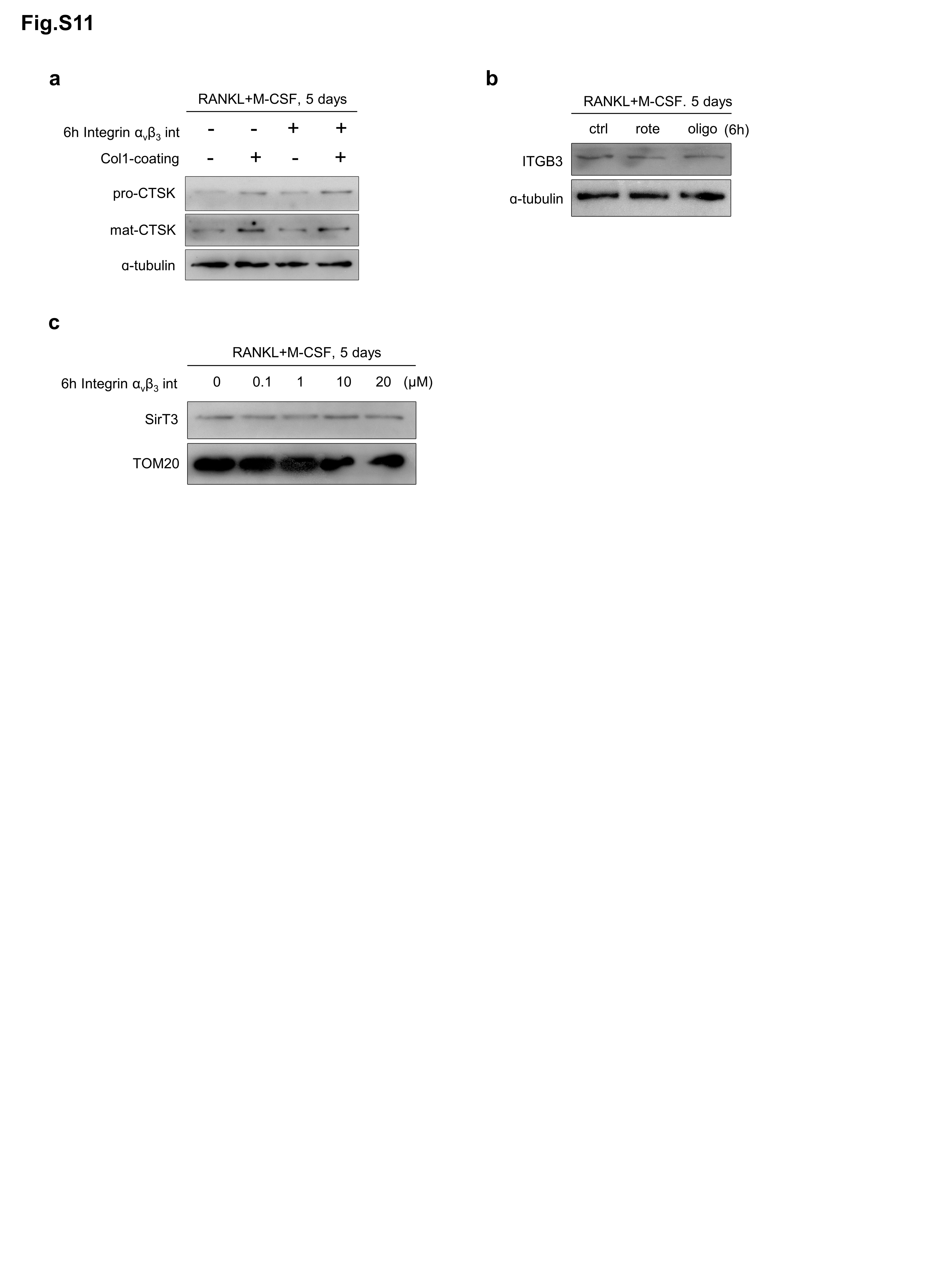

Supplement: Supplementary file 12 — Supplemental Figure 11 [file 41413_2024_360_MOESM12_ESM.tif]
